# A CPF-like phosphatase module links transcription termination to chromatin silencing

**DOI:** 10.1016/j.molcel.2024.05.016

**Published:** 2024-06-07

**Authors:** Eduardo Mateo-Bonmatí, Miguel Montez, Robert Maple, Marc Fiedler, Xiaofeng Fang, Gerhard Saalbach, Lori A. Passmore, Caroline Dean

**Affiliations:** 1 Cell and Developmental Biology, John Innes Centre, Norwich Research Park, Norwich NR4 7UH, UK; 2 Centro de Biotecnología y Genómica de Plantas, Universidad Polité cnica de Madrid (UPM), Instituto Nacional de Investigación y Tecnología Agraria y Alimentaria (INIA)/ CSIC, Pozuelo de Alarcón, Madrid 28223, Spain; 3 MRC Laboratory of Molecular Biology, Cambridge CB2 0QH, UK; 4 Biological Chemistry, John Innes Centre, Norwich Research Park, Norwich NR4 7UH, UK

## Abstract

The interconnections between co-transcriptional regulation, chromatin environment, and transcriptional output remain poorly understood. Here, we investigate the mechanism underlying RNA 3′ processing-mediated Polycomb silencing of *Arabidopsis FLOWERING LOCUS C* (*FLC*). We show a requirement for ANTHESIS PROMOTING FACTOR 1 (APRF1), a homolog of yeast Swd2 and human WDR82, known to regulate RNA polymerase II (RNA Pol II) during transcription termination. APRF1 interacts with TYPE ONE SERINE/THREONINE PROTEIN PHOSPHATASE 4 (TOPP4) (yeast Glc7/human PP1) and LUMINIDEPENDENS (LD), the latter showing structural features found in Ref2/PNUTS, all components of the yeast and human phosphatase module of the CPF 3′ end-processing machinery. LD has been shown to co-associate *in vivo* with the histone H3 K4 demethylase FLOWERING LOCUS D (FLD). This work shows how the APRF1/LD-mediated polyadenylation/termination process influences subsequent rounds of transcription by changing the local chromatin environment at *FLC*.

## Graphical abstract

**Graphical abstract:**
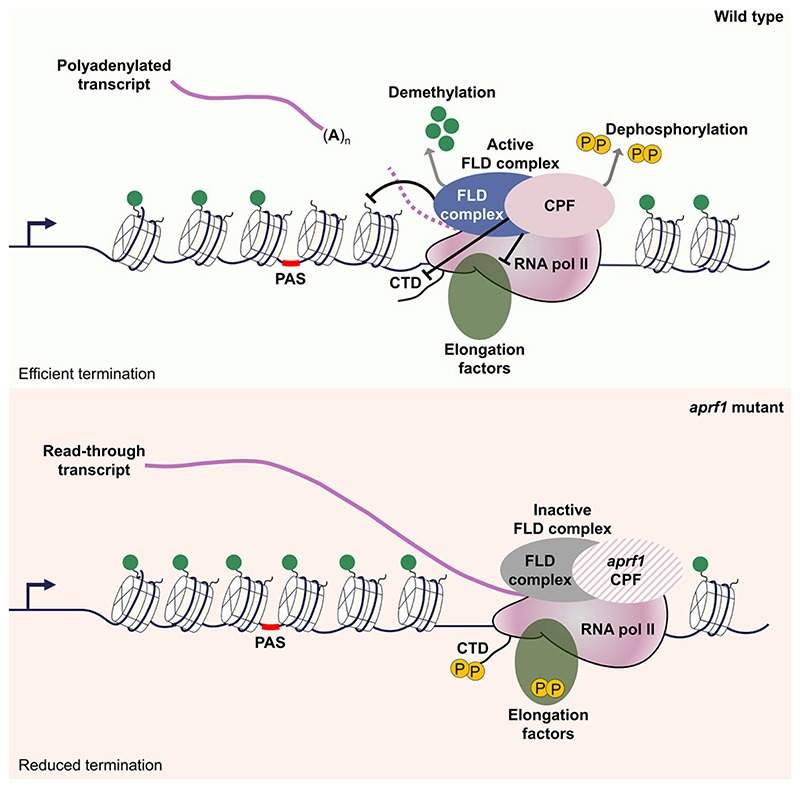

## Introduction

The relationship between chromatin and transcription in gene regulation is complex, involving feedback mechanisms that make the interaction difficult to dissect. For example, chromatin influences RNA polymerase II (RNA Pol II) processivity, i.e., the likelihood of transcription reaching the end of the gene, which can lead to alternative splicing or early termination. In turn, these co-transcriptional steps feed back to influence the chromatin state.^1–3^ One locus where this complexity has been studied in detail is *Arabidopsis FLC* (*FLOWERING LOCUS C*). *FLC* encodes a floral repressor, and quantitative variation in *FLC* transcription has been an important determinant in adaptation of *Arabidopsis* accessions to a wide range of climates. For instance, high *FLC* expression upon germination in autumn enables an over-wintering reproductive strategy, with winter-induced Polycomb silencing of *FLC* aligning flowering with spring.^4^ In contrast, low *FLC* expression through a developmentally induced Poly-comb silencing leads to a rapid-cycling reproductive strategy, allowing multiple generations per year in some climates.

There has been extensive analysis of the components regulating *FLC* transcription to understand the developmentally induced silencing. The first repressor identified was FCA (FLOWERING CONTROL LOCUS A), an RRM (RNA recognition motif)-containing RNA binding protein^5^ that directly binds to *FLC* antisense transcript *COOLAIR*, an association promoted by the presence of an R loop.^6^ Forward genetic screens identified additional repressors that include other RNA binding proteins, RNA 3′ processing factors and chromatin modifiers.^7^ These factors have been found to promote proximal termination of both *COOLAIR* in seedlings and sense *FLC* transcripts in the embryo.^8^ Suppressor genetics and proteomic analysis showed that these co-transcriptional activities function through three factors that associate with each other *in vivo*—FLD (FLOWERING LOCUS D; a H3K4 demethylase), LD (LUMINIDE-PENDENS; transcription factor IIS [TFIIS] domain protein), and SDG26 (a SET domain protein).^9,10^ These induce histone H3K4 demethylation across the locus to suppress *FLC*. SDG26 physically associates with FY/WDR33, a cleavage and polyadenylation specificity factor (CPSF; CPF in yeast) component, after cross linking^10^ and both reduce H3K4me1 and H3K36me3 accumulation at *FLC*. This antagonizes transcription, enabling a switch to Polycomb repressive complex 2 (PRC2)-mediated H3K27me3 accumulation, thus reducing transcriptional initiation and elongation rates.^11^ The *FLC* silencing mechanism thus involves co-transcriptional processes that mediate chromatin modifications, which in turn feed back to reinforce the co-transcriptional processing. The factors involved are all evolutionarily conserved and affect many genes in *Arabidopsis*,^12,13^ raising the possibility that the mechanism may be broadly relevant in gene regulation.

A key question that remains is how proximal termination delivers a changed histone environment that enables the PRC2 switch. In the work described here, we found a robust interaction of the FLD-LD-SDG26 complex with *Arabidopsis* APRF1 (ANTHESIS PROMOTING FACTOR 1), homologous to CPF phosphatase module component Swd2/WDR82. APRF1 also interacts with TOPP4 (TYPE ONE SERINE/THREONINE PROTEIN PHOSPHATASE 4), homologous to CPF phosphatase module component Glc7/PP1. The CPF phosphatase module dephosphorylates the RNA Pol II and its partners via the Glc7/PP1, an activity that promotes transcription termination through effects on RNA Pol II elongation/processivity.^14–16^ Close examination revealed that LD is structurally related to Ref2 in yeast and PNUTS in mammals. Ref2/PNUTS act in yeast/mammalian complexes as the regulatory subunit of the CPF phosphatase module.^15,17^ Using molecular and genetic tools, we show that APRF1-dependent RNA processing activities function in the same co-transcriptional pathway as the FLD-LD-SDG26 chromatin modifier complex to promote proximal termination of the antisense *COOLAIR* transcripts, alter *FLC* chromatin environment, and affect *FLC* transcriptional output. This chromatin environment reinforces proximal termination choice, thus providing the molecular feedback necessary to stably maintain a low transcription state.

This work describes the mechanism linking transcription termination/regulation of RNA Pol II and histone demethylation. APRF1, a structural component of a CPF-like phosphatase complex, directly links transcription termination with histone demethylase activity to alter the local chromatin environment and provide a mechanism resulting in graded repression of transcription. How this leads to the switch to Polycomb silencing was not resolved, as none of the proteomic experiments identified Polycomb components. Our accompanying paper describes the use of mathematical modeling and experiments to elucidate how the mechanism described here sets the level of productive (processive) transcription that promotes the digital switch to the Polycomb silenced state.^18^

## Results

### The FLD complex robustly associates with APRF1, the homolog of yeast Swd2

We previously reported that the histone demethylase homolog FLD^19^ associates with LD^20^ and SDG26^21^ *in vivo*; each tagged version of these three proteins enriched the other two partners in co-immunoprecipitation (coIP) experiments.^10^ Interestingly, each of these proteins also co-immunoprecipitated with anthesis promoting factor 1 (APRF1),^10^ a result recently confirmed in an independent analysis.^22^

**Figure 1:**
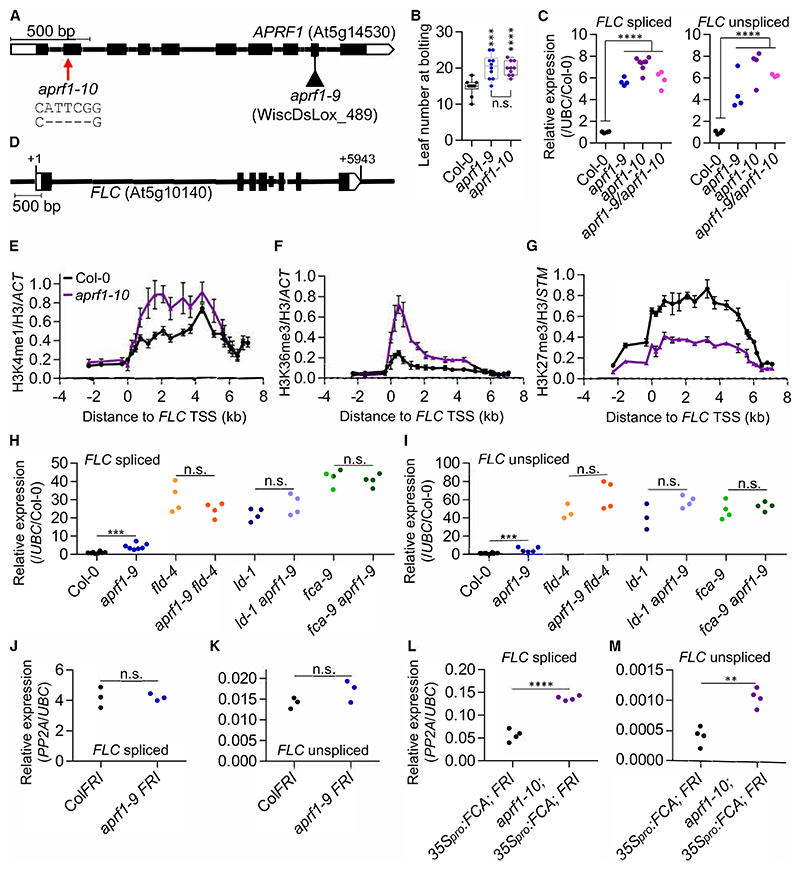
APRF1, a robust interactor of the FLD complex, functions genetically in the FCA pathway A) Architecture of the *APRF1* gene and illustration of the nature and location of the mutations studied in this work. Boxes indicate exons and lines introns. White boxes represent untranslated regions. The triangle represents a T-DNA insertion (*aprf1-9*); the red arrow points to the location of the CRISPR-Cas9-derived deletion (*aprf1-10*). B) Boxplot representing the leaf number at bolting of the wild-type Col-0 and both *aprf1* mutants. Each dot represents the score of a single plant. Boxes are delimited by the first (Q1, lower hinge) and third (Q3, upper hinge) quartiles. Whiskers represent Q1 –1.5 IQ (lower) and Q3 +1.5 IQ (upper), where IQ = Q3 – Q1. Horizontal bars represent the median of the values. C) Relative values of *FLC* spliced (left) and unspliced (right) in the wild-type Col-0, both *aprf1* mutants, and F_1_ *aprf1-9/aprf1-10* hybrid plants. Values were normalized to the housekeeping *UBC* gene and to Col-0. D) Schematic diagram showing *FLC* gene structure following the guidelines described for (A). +1 indicates the transcriptional start site (TSS). (E–G) ChIP analysis of H3K4me1 (E), H3K36me3 (F), and H3K27me3 (G) levels at *FLC* in Col-0 and *aprf1-10*. Numbers in x axis represent the distance in kilobases to the *FLC* TSS and numbers in the y axis correspond to relative enrichment of the corresponding histone mark. Each dot represents an amplicon. Values were normalized to H3 and to *ACT7* (for H3K4me1 and H3K36me3) or *STM* (for H3K27me3) and represent mean ± standard error of the mean (SEM). (H−M) Relative values of *FLC* spliced (H, J, and L), and unspliced (I, K, and M) in various genetic backgrounds. Values were normalized to the housekeeping gene *UBC* (H–M) and Col-0 (H and I) or *PP2A* (J–M). Asterisks indicate statistically significant differences to Col-0 (B, C, H, and I) and to C2 (*35S_pro_:FCA*g; *FRI*) (L and M) in a two-way Student’s t test (** *p* < 0.01, *** *p* < 0.001, and **** *p* < 0.0001). N.s. stands for not statistically different (*p* > 0.05). Scale bars: 500 bp in (A) and (D). Experiments were performed using 2-week-old seedlings grown in long days conditions with *n* ≥ 3 (C and E−M), where each replicate represents a pool of 10 to 15 seedlings (C and H–M) or 2.5 g of seedlings (E–G).

APRF1 is a WD40-repeat protein encoded by At5g14530. We obtained a transfer DNA (T-DNA) insertion line (WiscDsLox_489; *aprf1-9*; Kapolas et al.^23^) and analyzed the flowering time and *FLC* expression. Both *FLC* spliced and unspliced transcripts were significantly upregulated in the mutant line and, accordingly, *aprf1-9* plants were late flowering (Figures 1A−1C). The insertion interrupts the 9^th^ of 10 exons, so it was possible that this mutant retained some APRF1 function. To address this possibility, we designed a CRISPR-Cas9 transgene to generate a full knockout for *APRF1*. Using an sgRNA targeting the second exon and screening for edited plants, we found transgene-free T_2_ plants carrying a 5-nt deletion that creates an in-frame premature stop codon (Figure S1). This new allele was named *aprf1-10* and was as late flowering as *aprf1-9* (Figure 1B). We crossed *aprf1-9* to *aprf1-10* and analyzed *FLC* expression levels of the single and heterozygous mutants. All had similarly upregulated *FLC*, confirming their allelism and the role of APRF1 in *FLC* repression (Figure 1C).

### Swd2 function is functionally diverged in *Arabidopsis*

APRF1 is one of the two *Arabidopsis* orthologs of the *Saccharomyces cerevisiae* (*S. cerevisiae*) Swd2 (Figure S2A).^24^ Swd2 has been found to play a role in two very different complexes. One of these is the complex of proteins associated with Set1 (COMPASS), responsible for the co-transcriptional deposition of H3K4me3, where Swd2 promotes the interaction between Set1 and the RNA Pol II carboxy terminal domain (CTD).^25^ Additionally, Swd2 has been identified as a component of the phosphatase module of the CPF (CPSF in higher eukaryotes) and the associated with Pta1 (APT) complexes, which signal transcriptional termination.^26,27^ These apparently opposite roles of transcription in yeast motivated us to test whether the *Arabidopsis* orthologs *APRF1* (also known as *Swd2-like A/S2LA*) and *S2LB* had functionally diverged. *FLC* spliced and unspliced levels were assayed in an insertional allele of *S2LB* (Figure S2B) and the double mutant *aprf1-9 s2lb*. Opposite to *aprf1-9, s2lb* showed a significant reduction in *FLC* expression, while the double mutant had *FLC* levels indistinguishable from the *aprf1-9* single mutant (Figure S2C). We further analyzed the effects on *FLC* expression in a representative mutant of COMPASS activity, *atx1-2*, an insertion allele in *ARABIDOPSIS TRITHORAX 1* (*ATX1*), which encodes one of the methyltransferases. In line with the results observed for *s2lb*, both *FLC* spliced and unspliced were significantly downregulated in *atx1-2* compared with Col-0 (Figure S2D). These results perfectly match with previous work reporting *FLC* downregulation in other COMPASS mutants defective in components such as WDR5 (WD-40 repeat-containing protein 5)^28^, RBL(retinoblastoma-binding protein-like), or ASH2R (ARABIDOPSIS ASH2 RELATIVE).^29^ FLD-mediated repression of *FLC* requires H3K4me1 removal.^10,12^ Considering the tight link between the FLD complex and APRF1, we performed chromatin immunoprecipitation coupled with quantitative PCR (ChIP-qPCR) to quantify H3K4me1 at *FLC*. We found higher levels of H3K4me1 across the locus in *aprf1-10* compared with Col-0 (Figure 1E), matching results in *ld-1* and *fld-4* (Figure S2E)^10^ or genome-wide.^12^ In contrast, *s2lb* plants showed slightly lower levels of H3K4me1 than the Col-0 (already low), in line with COMPASS dysfunction (Figure S2E). The contrasting phenotypes shown by *s2lb* and *aprf1* suggest that, in *Arabidopsis*, the ancestral Swd2 may have sub-functionalized, with S2LB working through the COMPASS complex and therefore activating *FLC*, and APRF1 working with the 3′ processing machinery to repress the locus (Figure S2F).

H3K4me1 binds SDG8,^30^ a histone methyltransferase that deposits H3K36me3, an active histone modification, which is mutually exclusive to the repressive PRC2-deposited H3K27me3 at *FLC*.^31^ Consistent with *FLC* upregulation and the increased H3K4me1 levels in *aprf1-10*, ChIP-qPCR analyses also showed that H3K36me3 and H3K27me3 were upregulated and downregulated, respectively. Thus, APRF1 has a role in establishing a silent chromatin state at *FLC* (Figures 1F and 1G).

### APRF1 functions downstream of FCA

Working genetically upstream of FLD functionality, FCA promotes proximal termination on both strands of *FLC*.^8,32^ To obtain genetic evidence of the relationship between *APRF1* and *FLD, LD*, or *FCA*, we crossed *aprf1-9* with mutants in subunits of the FLD complex, such as *fld-4* or *ld-1*, and the core cosmponent of the pathway *fca-9. FLC* levels in the double mutants revealed an epistatic relationship between *FLD, LD*, or *FCA* and *APRF1* (Figures 1H and 1I), further demonstrating the genetic connection between *APRF1* and the *FLC* repression machinery. To study the effects of loss of *APRF1* in a high transcriptional environment, but without perturbing the FCA pathway, we introgressed a functional *FRIGIDA* (*FRI*)^33^ into *aprf1-9*. FRI functions as an *FLC* transcriptional activator and, like other anti-terminators,^34^ promotes distal polyadenylation of both *FLC* and *COOL-AIR*, thus antagonizing the co-transcriptional repression mechanism.^8^ Levels of *FLC* in *aprf1-9 FRI* were the same as Col*FRI*, likely due to an overwriting effect of *FRI* compared with the relatively modest *FLC* upregulation of *aprf1-9* (Figures 1J and 1K). Finally, we previously generated a sensitized transgenic system, called C2, in which the chromatin of *FLC* is silenced even in the presence of an active *FRI* by the overexpression of the *FCA* through the transgene *35S_pro_:FCA*g.^7,35^ Mutations affecting FCA downstream processes compromise the FCA-mediated *FLC* chromatin silencing. Introgression of *aprf1-10* into the C2 background resulted in a significant release of *FLC* repression, demonstrating a role for APRF1 downstream of FCA (Figures 1L and 1M).

### APRF1 reciprocally interacts with LD, the plant homolog of the phosphatase regulatory subunit Ref2/PNUTS

**Figure 2:**
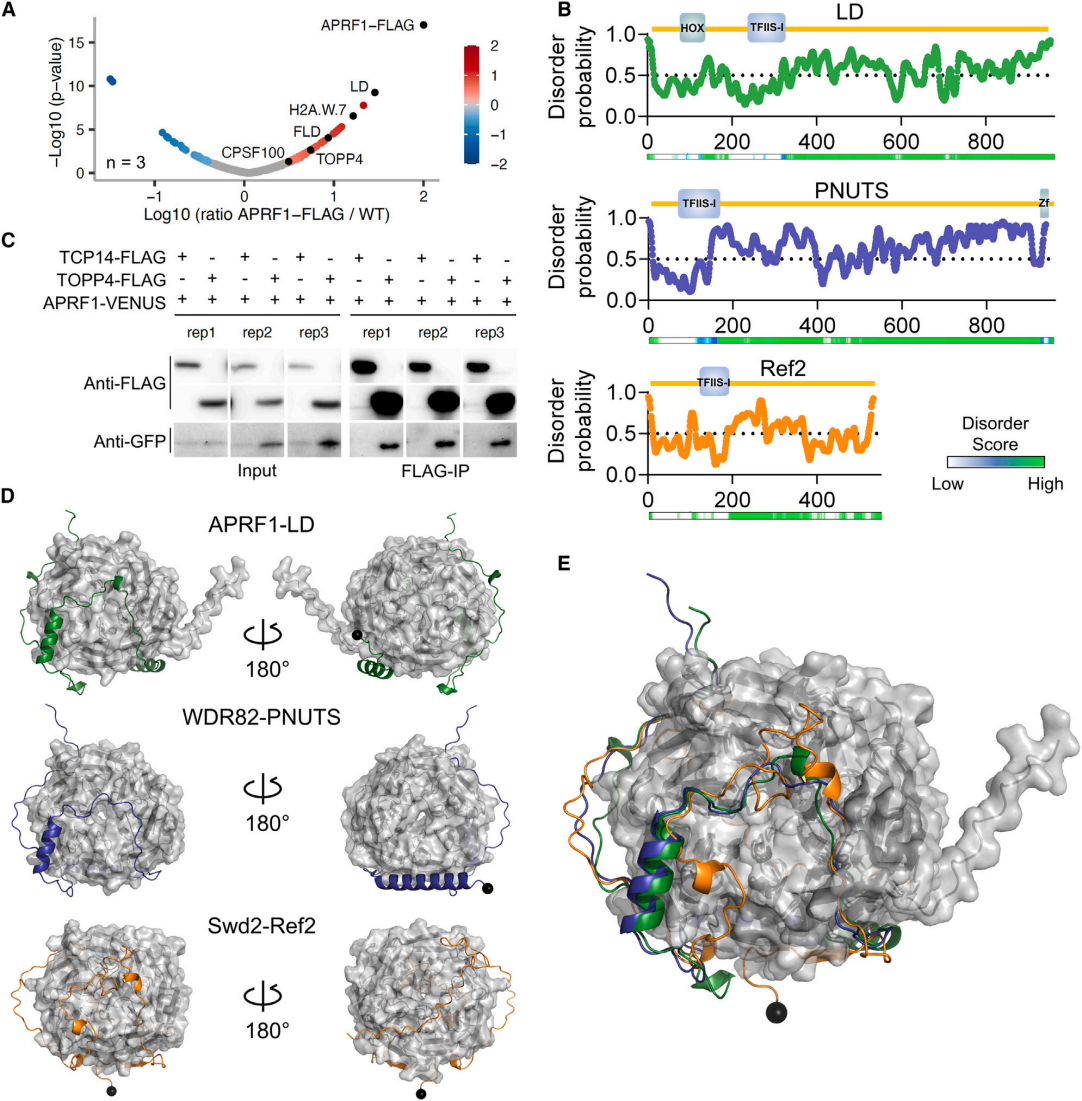
LD-APRF1-TOPP4 form a plant CPSF-like phosphatase module (A) Volcano plot showing the relative protein abundance in log-10 scale ratio of immunoprecipitated samples from APRF1-3xFLAG to control Col-0 samples, analyzed in triplicate. Each replicate consists of 2 g of 10-day-old seedlings. Red dots highlight proteins enriched in the APRF1-3xFLAG samples. APRF1-FLAG, LD, H2A.W.7, FLD, TOPP4, and CPSF100 are shown as black dots. *p* values were obtained based on hypothesis testing by t test. More detail in STAR Methods. (B) Schematic representation of protein size, annotated domains, disorder probability, and disorder score for LD, PNUTS (*Homo sapiens*), and Ref2 (*Saccharomyces cerevisiae*). Individual amino acid score for disorder probability were obtained with the online Protein DisOrder prediction System (PrDOS) and plotted using GraphPad. Disorder scores were obtained by D^2^P^236^ and shown in a color scale. (C) Co-immunoprecipitation results obtained in transiently transformed leaves of *N. benthamiana* with APRF1-mVENUS, TOPP4-3xFLAG, and the control line TCP14-FLAG. Three replicates of the same experiment are shown. Full blot details in Figure S8. (D) AlphaFold2 predictions of complexes between APRF1-LD, WDR82-PNUTS, and Swd2-Ref2. WD40 orthologs are shown in gray surface representation, whereas TFIIS orthologs are shown in cartoon; the N-terminal amino acid of the predicted TFIIS proteins are denoted by black spheres. (E) Overlay of the three predictions shown in (D).

To further characterize the role of APRF1 in *FLC* repression, we performed crosslinked nuclear immunoprecipitation and mass spectrometry (CLNIP-MS) using 10-day-old seedlings of a FLAG-tagged version of APRF1.^22^ We found that the top hit was LD, thus confirming their reciprocal interaction (Figure 2A; Table S1). Consistent with ours^10^ and other reports,^22^ among the top hits we also found FLD (Figure 2A). Interestingly, one of the highest hits was the histone H2A.W.7, a variant exclusively found on constitutive heterochromatin,^37,38^ suggesting a generic role for APRF1 in co-transcriptional gene repression. Given this robust APRF1-LD interaction, we considered whether LD could be the homolog of one of the yeast Swd2 partners in the CPF or the APT phosphatase modules.^26,27^ Ref2 was an interesting candidate because both Ref2 and LD are highly unstructured proteins (Figure 2B). Ref2 is key for the interaction between CPF and the RNA Pol II and is a phosphatase regulatory subunit, providing substrate specificity to the phosphatase Glc7.^15,39,40^ Ref2 and its putative metazoan ortholog PNUTS are largely disordered proteins, apart from a TFIIS motif at the N-terminal region of the protein that consists of a compact four-helix bundle (Figure 2B). Intriguingly, despite its apparent key role in an otherwise highly conserved 3′ processing machinery, Ref2 shows no obvious homology with an *Arabidopsis* protein. We then performed BLAST searches to find potential orthologs in *Arabidopsis* for PNUTS.^41^ BLAST algorithms showed the best hit for PNUTS in *Arabidopsis* is LD (Figure S3). We aligned the sequences of Ref2, PNUTS, and LD, and although the degree of conservation was low (Figure S4), it improved slightly when aligning only LD and PNUTS (Figure S5). Nevertheless, all of them share similar features—a TFIIS domain in the N terminus in an overall highly unstructured protein (Figures 2B and S4B−S4D). PNUTS is known to interact with WDR82, a bona fide homolog of APRF1 (Figure S6), and provides substrate specificity to PP1 phosphatases like Glc7. We then searched for phosphatases among the APRF1 interactors, finding a highly significant interaction with TOPP4 (Figure 2A), a protein with very high homology to Glc7 and PP1 (Figure S7) and proven phosphatase activity *in vivo*,^42^ and C-terminal domain phosphatase-like 3 (CPL3), homolog to yeast FCP1, whose role in activating *FRI* complex activity has been reported.^43^ We have experimentally validated the interaction between APRF1 and TOPP4 *in planta* using transient coIP in *Nicotiana benthamiana* leaves of *TOPP4_pro_:TOPP4-3xFLAG* and *APRF1_pro_:APRF1-mVENUS* (Figure 2C). The IP-MS experiment also found a significant interaction between APRF1 and CPSF100, a structural component of the CPSF homolog of the yeast Cft2. In summary, our results suggest that APRF1/LD/TOPP4 form a plant equivalent of the yeast (Swd2/Ref2/Glc7) and human (WDR82/PNUTS/PP1) CPF phosphatase modules.

Further exploring these parallels, we carried out an *in silico* prediction using AlphaFold2^44^ of the interaction between APRF1-LD and their yeast and human counterparts (Figure 2D). Despite the low sequence homology, predictions support conservation of structural features and contact points between APRF1/Swd2/WDR82 and LD/Ref2/PNUTS, thus supporting their functional equivalence (Figure 2E). Taken together, the robust immunoprecipitation of LD and TOPP4 by APRF1, and the structural parallels between LD, Ref2, and PNUTS, suggest that LD, APRF1, and TOPP4 form a CPF or CPF-like phosphatase module in *Arabidopsis*.

### FLD complex and RNA Pol II co-occupy *FLC* chromatin independently of FCA function

**Figure 3:**
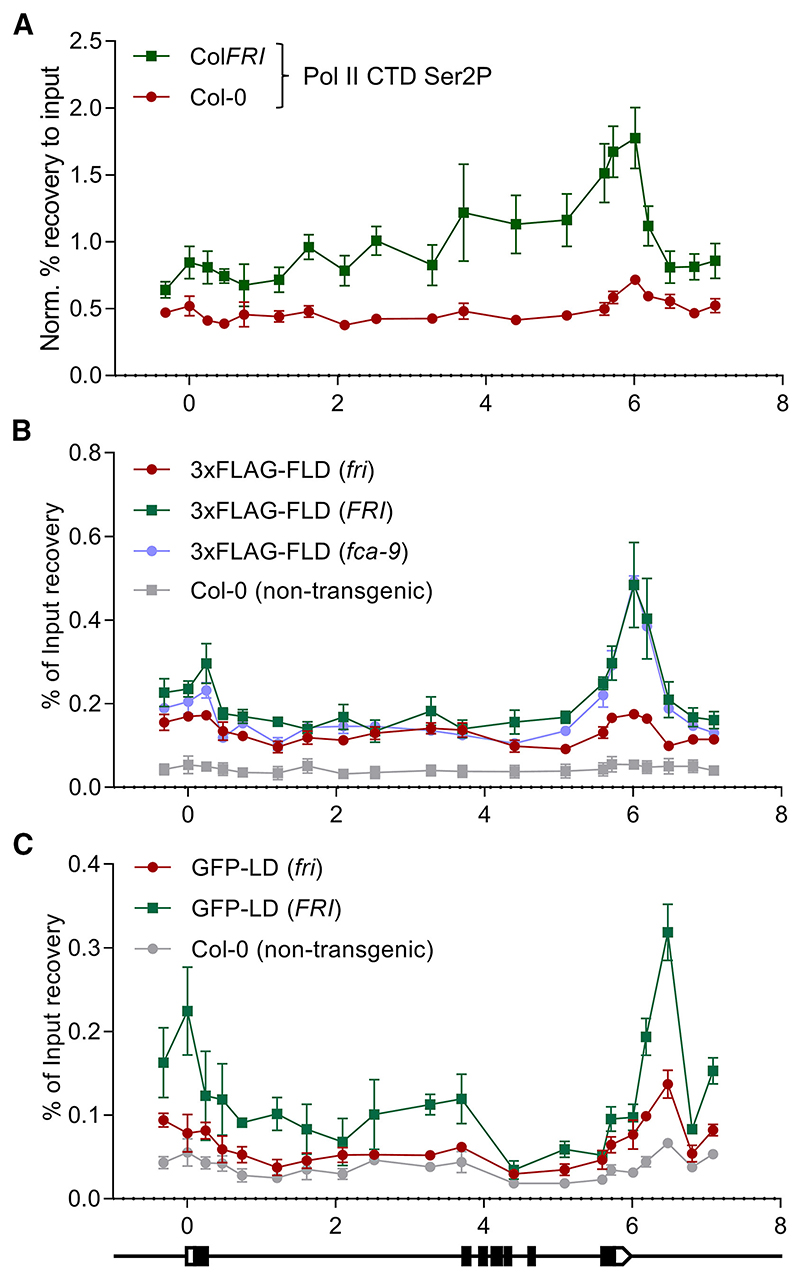
ChIP-qPCR co-occupancy profiles indicate that FLD and LD work co-transcriptionally to control *FLC* transcription (A) Elongating RNA Pol II (Ser2P) ChIP profiles over *FLC* in a high (Col*FRI*) and low (*fri* or Col-0) transcriptional background. (B) ChIP binding profile over *FLC* of the *fld-4 FLD_pro_:3xFLAG-FLD* transgenic plants in different genetic backgrounds. (C) ChIP binding profile over *FLC* of the *ld-1* LD_*pro*_*:GFP-LD* transgenic plants in different genetic backgrounds. *FLC* gene structure following the indications for Figure 1D. All the experiments were done with 2-week-old seedlings. Dots and error bars represent mean ± SEM of three replicates. Each replicate consists of 2.5 g of seedlings. (A) Results expressed in % recovery to input values normalized to the promoter of the housekeeping gene *ACT7* as in Mikulski et al.^47^. Results in (B) and (C) are expressed in % recovery to input.

Available genome-wide data show FLD binding correlates with actively transcribed genes and elongating RNA Pol II CTD phosphorylated at Ser2 or Ser5.^12^ Similarly, the *Arabidopsis* FLD paralog LDL3 has been recently reported to work co-transcriptionally to remove H3K4me2.^45^ RNA Pol II occupancy in the *Arabidopsis* genome often shows peaks near transcription termination sites (TTSs), potentially linked to slow co-transcriptional termination events.^11,46^ We found that this was the case for *FLC* in Col*FRI* vs. Col-0 (Figure 3A), and has been shown to be the case in *fca-9* or *fld-4*.^11^ To address co-occupancy with FLD, we performed ChIP-qPCR using a transgenic FLAG-tagged FLD, with and without the transcriptional activator FRI, and non-transgenic control plants. Agreeing with the reported data,^12^ FLAG-FLD showed high enrichment at the 3′ region of *FLC* in an *FRI* genotype compared with Col-0 (*fri*), with the latter close to the background signal (Figure 3B). To rule out the possibility that this enrichment was an effect of the transcriptional activator FRI and not the high transcription itself, we introgressed the FLAG-FLD transgene into the *fca-9* background. FLD enrichment at the 3′ end of *FLC* in *fca-9 FLAG-FLD* was indistinguishable from *FRI FLAG-FLD*, confirming FLD enrichment primarily associated with transcriptional activity (Figure 3B). *FLD* functions genetically downstream of *FCA*,^9^ so it was interesting that FLD-Pol II co-occupancy association was not affected by *fca-9*. Thus, even when FLD is located at *FLC*, it cannot function properly without FCA-mediated 3′ processing of the nascent transcript.

We introgressed a GFP-tagged LD^10^ into Col*FRI* to create a line with high *FLC* transcription and performed ChIP-qPCR experiments. LD enrichment, peaking at the *FLC* TTS, was only detected in the line where *FLC* transcription is high (Figure 3C). The shared enrichment of elongating Pol II, FLD, and LD is consistent with an *in vivo* association of the transcriptional machinery and the FLD complex, as suggested by genome-wide data.^12^ Thus, we propose that FLD and LD associate with RNA Pol II and, therefore, work co-transcriptionally, fitting with LD working as a CPF component equivalent to Ref2 or PNUTS.

### Inefficient termination in *aprf1* mutants leads to transcriptional readthrough

**Figure 4:**
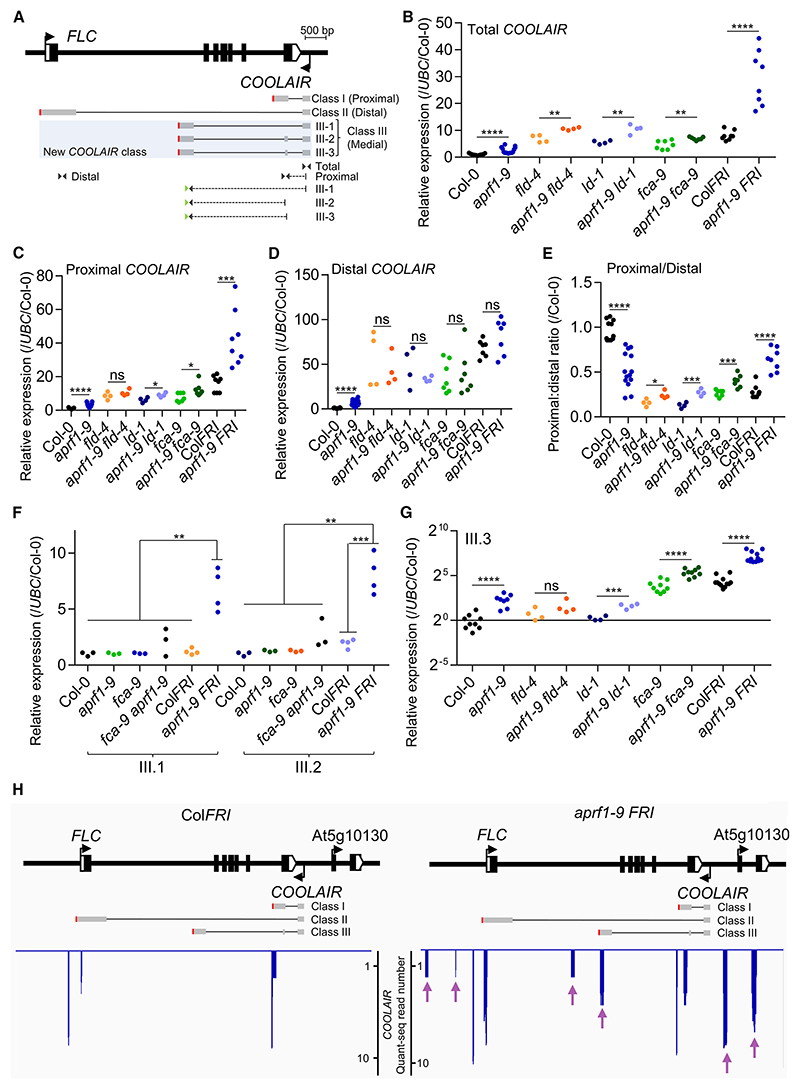
Mutations in *APRF1* trigger *COOLAIR* upregulation and an increase in a new medially polyadenylated *COOLAIR* isoform (A) *FLC* architecture following the representation of Figure 1D with indication of different *COOLAIR* isoforms in gray. For simplicity, classes I and II (proximal and distal) are each represented by one isoform. The new *COOLAIR* class III isoforms are highlighted in pale blue. Triangles represent primer pairs (not drawn to scale) used to measure the relative abundance by RT-qPCR. Dashed lines indicate primers spanning two *COOLAIR* exons. Green triangles indicate the primer used for *COOLAIR* class III retro-transcription. Red vertical lines represent polyadenylation sites. Scale bars, 500 bp. (B–D) Relative expression analyses of (B) total *COOLAIR*, (C) proximal *COOLAIR*, and (D) distal *COOLAIR* in different genotypes. (E) *COOLAIR* proximal-to-distal ratio in different genotypes. (F and G) Relative expression analyses of *COOLAIR* classes (F) III.1 and III.2, and (G) III.3. Each dot represents a biological replicate analyzed in triplicate. Each replicate consists of a pool of 10 to 15 seedlings. Expression values were normalized to the *UBC* gene and to Col-0. Asterisks indicate statistically significant differences to the indicated genotypes in a Student’s t test (* *p* < 0.05, ** *p* < 0.01, *** *p* < 0.001, and **** *p* < 0.0001). N.s. stands for not statistically different. All the experiments were performed in 2-week-old seedlings grown in long days conditions. (H) *COOLAIR* strand Quant-seq results of Col*FRI* and *aprf1-9 FRI* seedlings. *FLC* locus is represented as in (A). The blue spikes indicate reads supporting a polyadenylation site. Purple arrows point to clusters of reads present in *aprf1-9 FRI* but absent in Col*FRI*.

Previous work on the FCA pathway found that most of the factors involved directly affected *FLC* antisense transcripts (*COOLAIR*).^7^ To pursue a potential role of APRF1 in transcriptional termination, we analyzed the transcription and processing of *COOLAIR*. In contrast to *FLC* levels, which did not show any significant difference (Figures 1J and 1K), total *COOLAIR* levels were upregulated in double mutants containing *aprf1-9* (Figures 4A and 4B). This was particularly striking for the *aprf1-9 FRI* combination. *COOLAIR* transcripts are polyadenylated at many sites, with major clusters at proximal sites (class I) and distal sites coincident with the *FLC* promoter (class II; Figure 4A).^35^ An increase in proximal *COOLAIR* (Figure 4C) and no change in distal *COOLAIR* were found in *aprf1-9 FRI* (Figure 4D), with the proximal/distal ratio increased in *aprf1-9* double mutant combinations compared with single mutants (Figure 4E), unlike other mutations affecting the FCA pathway.^48^ However, as with the *Arabis alpina FLC* ortholog (*PEP1*),^49^ we detect low-abundance spliced *COOLAIR* transcripts polyadenylated around a medial site (F. Liu and C.D., unpublished data), which we term *COOLAIR* class III (Figure 4A). This isoform was significantly upregulated, specifically in *aprf1-9 FRI* (Figure 4F), with one specific spliced variant *COOL-AIR* class III.3 becoming the most abundant isoform, enriched in high transcription situations such as *fca-9* or Col*FRI* (Figures 4G, S9A, and S9B). In line with the proposed divergent roles and consistent with the *FLC* sense expression profile (Figures S2C and S2D), *s2lb* and *atx1-2* mutants showed significantly lower levels of all the *COOLAIR* isoforms compared with Col-0 (Figure S9C). The high levels of the *COOLAIR* class III isoform seemed likely to reflect inefficient polyadenylation/transcription termination at the proximal site. To analyze the polyadenylation site (PAS) choice in an unbiased and strand-specific manner in these two genotypes, we carried out a Quant-seq analysis of Col*FRI* and *aprf1-9 FRI* to detect the mRNA 3′ end. There were only a small number of reads on the *FLC* (sense) strand, consistent with transcriptional readthrough (Figure S10). However, for *COOLAIR* the differences were large, with many reads indicating use of the medial poly A site (class III), distal *COOLAIR* readthrough, and alternative *COOLAIR* transcriptional starts (Figures 4H and S10). To determine whether the effects were specific to loss of APRF1, we performed Quant-seq analysis of *fld-4* compared with the wild-type Col-0 (Figure S11). *FLC* sense transcripts were qualitatively the same, just quantitatively upregulated in *fld-4* (Figures S11A and S11B). The few reads corresponding to *COOLAIR* were insufficient to make any conclusions (Figure S11B), so a library enrichment was performed using baits encompassing the 20-kb *FLC* genomic region (see STAR Methods). As a control, we also performed this bait enrichment on the Col*FRI, aprf1-9 FRI* libraries (Figure S12). No medial poly A site (class III) were found in Col-0 or *fld-4* (Figures S11C and S11D), suggesting that the termination defects are APRF1-specific and that the FLD downstream function is not involved in the termination process.

Both Quant-seq datasets revealed more than two hundred commonly misregulated genes in *aprf1-9 FRI* and *fld-4* compared with the corresponding wild-type strain (Figures S13A and S13B; Tables S2 and S3). Among the genes upregulated in *fld-4*, and even more on those commonly upregulated in both mutant backgrounds, we observed a generalized shift from proximal-to-distal PAS choice in *aprf1-9 FRI* compared with Col*FRI* (Figures S13C–S13E). We also found Quant-seq signals compatible with readthrough or inefficient proximal polyadenylation in other genes (Figures S13F and S13G). Taken together, our Quant-seq analyses suggest that *APRF1* loss leads to inefficient transcriptional termination at many loci in the *Arabidopsis* genome.

**Figure 5:**
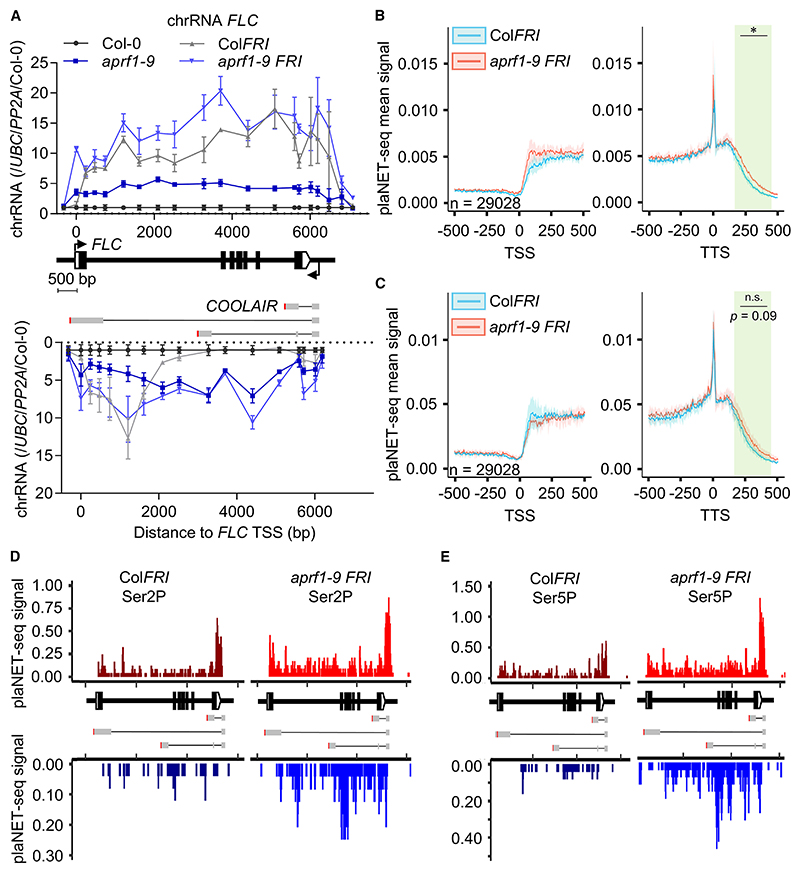
*COOLAIR* transcriptional readthrough correlates with changed phosphorylation of the RNA Pol II carboxy terminal domain (A) Chromatin-bound RNA levels of *FLC* and *COOLAIR* in *aprf1-9* mutants, with and without functional *FRI*. Data were normalized to *UBC* and *PP2A* and are shown as fold-change to wild-type Col-0. Dots correspond to amplicons of the *FLC* (upper chart) and *COOLAIR* (bottom chart) transcripts, represented as mean ± SEM of three biological replicates. Each replicate consists of 2.0– 2.5 g of seedlings. (B and C) plaNET-seq metaplots at TSS and TTS of Col*FRI* and *aprf1-9 FRI* using Ser2P (B) and Ser5P (C) antibodies, analyzed in triplicate. Each replicate consists of 3 g of 10-day-old seedlings. Asterisk indicates statistically significant differences between *aprf1-9 FRI* and Col*FRI* in a one-way ANOVA with multiple comparisons (* *p* < 0.05). (D and E) plaNET-seq profiles over the *FLC* locus of Col*FRI* and *aprf11-9 FRI* using Ser2P (D) and Ser5P (E) antibodies. Upper and bottom charts show plaNET-seq profiles with merged replicates, corresponding to *FLC* and *COOLAIR* strands, respectively.

To further investigate co-transcriptional changes, we analyzed chromatin-bound RNA (chRNA) of Col-0, *aprf1-9*, Col*FRI*, and *aprf1-9 FRI* at *FLC. FLC* (sense) chRNA levels of *aprf1-9* were higher than Col-0, as *aprf1-9* compromises *FLC* repression. PCR amplicons for sense *FLC* levels in Col*FRI* and *aprf1-9 FRI* gave similar values at the 5′ and 3′ ends of the locus, but there were differences in the central region, pointing to the complex effects of APRF1 on *FLC* Pol II processivity (Figure 5A). However, the most striking differences were on the *COOLAIR* strand (antisense) (Figure 5A). RNA was detected in regions corresponding to *COOLAIR* class I and class II in Col*FRI*, but in *aprf1-9* genotypes (*fri* and *FRI*) this signal extended to regions that cover both *COOLAIR* class I and class III transcripts. This likely represents antisense transcriptional readthrough, supporting a role for APRF1 as part of the RNA Pol II termination machinery (Figure 5A).

### APRF1 affects RNA Pol II CTD Ser2/5 phosphorylation

Different mechanistic models, not mutually exclusive, have been proposed for transcription termination.^14,50^ Current thinking based on studies in different organisms is that CPF-RNA Pol II recognition of PAS triggers the nascent RNA cleavage. This generates a free 5′ end on the cleaved RNA, which is a substrate for 5′−3′ ribonucleases such as XRNs (5′−3′ exoribonucleases) that degrade the nascent transcript and dislodge RNA Pol II from the chromatin.^14,17^ Transcription of the PAS also triggers a conformational change in RNA Pol II, potentially driven by dephosphorylation of the CTD and/or co-factors, which slows down RNA Pol II. To ascertain whether *aprf1* mutants affect RNA Pol II CTD modifications, we carried out ChIP-qPCR experiments using antibodies targeting the RNA Pol II CTD phosphorylated residues Ser2, Ser5, and Tyr1, comparing *aprf1-10* with Col-0. The phosphorylated RNA Pol II was detected at higher levels in *aprf1-10*, consistent with the locus being more actively transcribed (Figure S14A). We repeated our analyses comparing *ld-1* and the double mutant *aprf1-9 ld-1*, given that both genotypes have similar *FLC* RNA levels (Figure 1H). RNA Pol II Ser2P and Tyr1P levels were higher than Col-0 but identical between the single and the double mutant (Figure S14B). However, Ser5P levels were higher in the *ld-1 aprf1-9* plants, indicating a role for APRF1 in RNA Pol II CTD Ser5 dephosphorylation.

Because our previous analyses revealed that levels of *COOL-AIR* class III (Figures 4F−4H) and transcriptional readthrough (Figure 5A) were particularly clear in a *FRI* background without affecting the overall *FLC* expression levels (Figures 1J and 1K), we also performed ChIP-qPCR experiments using the RNA Pol II CTD antibodies for Col*FRI* and *aprf1-9 FRI*. RNA Pol II Ser2P and Tyr1P levels were identical, while there was a slight increase in RNA Pol II Ser5P toward the 5′ end of the locus (Figure S14C). In order to obtain more sensitive, genome-wide, and strand-spe-cific information on the effects of *APRF1* in this background, we carried out plant native elongating transcript sequencing experiments (plaNET-seq)^51^ using the native CTD phosphorylated residues Ser2P and Ser5P. We observed a statistically significant generalized readthrough in *aprf1-9 FRI* using Ser2P, and differences using Ser5P that were not significant (Figures 5B and 5C). At the *FLC* locus, and in striking contrast to the low-resolution and not-strand-specific ChIP experiments, plaNET-seq revealed an increase in Ser2P in *aprf1-9 FRI* in the (*FLC*) sense strand compared with Col*FRI*, with a higher peak at the 3′ region of the locus, in agreement with *aprf1-10* ChIP results (Figure 5D). The *COOLAIR* strand showed greater differences, with *aprf1-9 FRI* having higher levels overall than Col*FRI* and a sharp accumulation close to the class III polyadenylation site (Figure 5D). Ser5 phosphorylation differences were even bigger, with an obvious accumulation on both strands and increased Ser5P near the class III polyadenylation site (Figure 5E). These results could suggest a role for the APRF1 termination complex in removal of RNA Pol II Ser2P/Ser5P at *FLC*. However, we cannot rule out the possibility that the CTD hyperphosphorylation we observe is an indirect effect derived from an hyperphosphorylation of elongation factors such as SPT5.^17^

## Discussion

The study of developmental timing in plants has led to mechanistic dissection of chromatin-silencing mechanisms at the gene encoding the *Arabidopsis* floral repressor FLC. Since quantitative variation of *FLC* expression affects the reproductive strategy of *Arabidopsis*, any molecular variation can be subject to strong evolutionary selection. *FLC* is thus an excellent system to dissect RNA processing, chromatin regulation, and their interconnections and molecular feedbacks that generate low and high transcriptional states that underpin adaptively important variation in transcriptional output.

**Figure 6:**
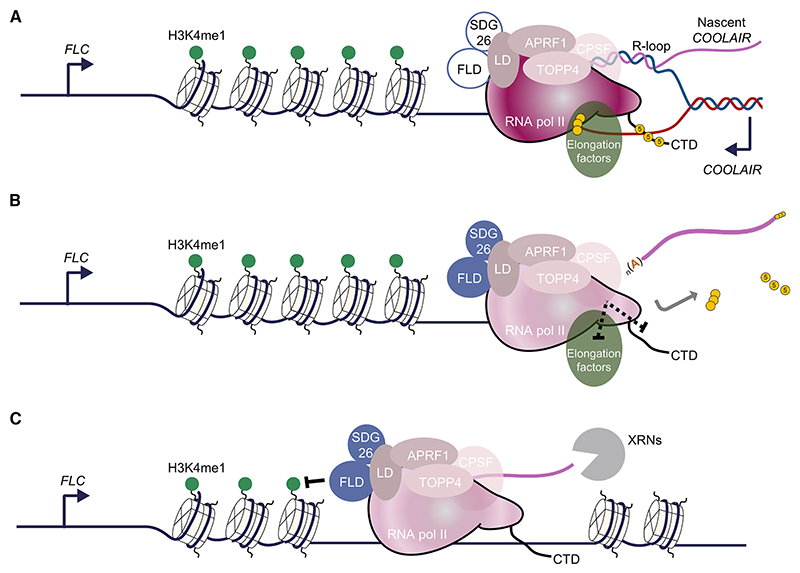
Proposed model for transcription-mediated chromatin silencing (A) Open *FLC* chromatin represented by white nucleosomes and marked with H3K4me1 (green circles) is actively transcribed by RNA Pol II machinery (solid maroon), which carries a non-active FLD complex (blue circle) as well as the 3′ end-processing machinery (CPSF), including the phosphatase module formed by APRF1-LD TOPP4, and elongation factors (green oval). Pol II CTD and elongation factors harbor some posttranslational modifications, including phosphorylation (yellow circles). Nascent *COOLAIR* forms an R-loop in the 3′ end of the locus.^6,52^ (B) Formation of the R-loop stimulates the 3′ end-processing machinery to terminate transcription at the proximal PAS. This termination is also signaled by the phosphatase module to Pol II via dephosphorylation of either elongating factors or the CTD or both (dashed line). PAS recognition triggers a conformational change on RNA Pol II, illustrated by a solid-to-pale maroon color change, which also activates FLD (now solid blue circle). (C) After *COOLAIR* is released, RNA Pol II continues transcribing an uncapped transcript that is the substrate of 5′−3′ exoribonucleases (XRNs). During this non-productive transcription, the FLD complex co-transcriptionally removes H3K4me1 marks from nucleosomes, creating a less processive chromatin environment for subsequent rounds of transcription.

This work identifies the importance of APRF1, a component of RNA Pol II termination machinery, in *FLC* regulation. We identify LD, a protein characterized as a flowering regulator nearly 20 years ago,^20^ as structurally related to yeast Ref2 and metazoan PNUTS proteins. Given its interaction partners, LD thus acts as a bridge between chromatin modifiers and the RNA Pol II machinery (Figure 6A). We propose that actively transcribed *FLC* chromatin is enriched with H3K4me1, which promotes RNA Pol II processivity, i.e., the likelihood of transcription reaching the end of the gene. Any pause in RNA Pol II functioning, for example, coincident with the formation of the R-loop during *COOLAIR* transcription, would stimulate the 3′ processing machinery (carried along in the RNA Pol II supercomplex) to polyadenylate the transcript at the proximal site. PAS recognition would signal activation of the APRF1-phosphatase module and trigger a conformational change on RNA Pol II (as proposed by Carminati et al.^15^) to activate FLD function (Figure 6B). RNA Pol II downstream of the PAS would proceed slowly due to the conformational change, causing FLD to remove H3K4me1 co-transcriptionally until the RNA Pol II is terminated by 5′−3′ XRNs (Figure 6C). This would create a less processive chromatin environment for subsequent rounds of transcription. Each round of proximal polyadenylation-termination-H3K4me1 removal would reinforce the next round of transcription, creating an intrinsic feedback loop in the mechanism.^18^ Therefore, we propose that transcription termination events contribute to the definition of chromatin domains around genes, preventing future transcriptional readthrough.

Intriguingly, our chRNA data detected very clear readthrough effects on the antisense (*COOLAIR*) strand, but also in certain parts of the sense (*FLC*) transcription unit when *APRF1* was disrupted. We thus envisage that this mechanism would operate on transcripts from both strands of *FLC* to first establish and then maintain the transcriptionally silenced state, with the conserved APRF1-LD machinery central to that chromatin silencing mechanism. Proximal termination of *FLC* sense transcription during early embryo development is associated with the establishment of the silenced state.^8^ Proximally polyadenylated sense transcripts do not accumulate in seedling tissue, but it is possible that these are particularly sensitive to RNA degradation pathways.

A question that arises is whether the CPF phosphatase module functioning at *FLC* associates with the majority of transcribing RNA Pol II or whether it provides a specialized function on *FLC*. Clearly, some parts of the genome are differentially enriched in co-transcriptional regulators. For example, *Arabidopsis* genome-wide data indicate that FLD shows a clear enrichment at sites of convergent transcription.^12^ CPF factors have also been shown to resolve DNA transcription/replication conflicts in plants and humans.^53,54^ Multiple protein phosphatase complexes have been described to promote transcription termination in eukaryotes. The Integrator-PP2C complex, which comprises 14 subunits (INT1-to-14) with limited conservation in plants,^55^ participates in both coding and non-coding termination.^56,57^ Only five of the INT subunits have clear *Arabidopsis* homologs (INT3/4/7/9/11), with a role in small nuclear RNA (snRNA) processing,^58^ but none were detected as an APRF1 interactor. The phosphatase module of the CP(S)F/APT and, more recently, the restrictor complex have been reported to play a role in transcriptional termination of mRNAs and non-coding RNAs, respectively.^59–61^ Homologs of APRF1 (Swd2 and WDR82) are constitutive members of the CPF/CPSF and restrictor complexes,^16,26,59–63^ but despite several zinc-finger proteins being among the APRF1 interactors, none appear to be equivalents of ZC3H4 as part of a plant restrictor complex (Table S1). Yeast CPF/APT phosphatase modules have other constitutive components, such as Pta1, Pti1, and Ssu72, with known orthologs in *Arabidopsis* (ESP4,^64^ Cstf64,^35^ and Ssu72^65^), but, again, these were not found to be APRF1 or FLD complex interactors. Thus, the data we present indicates that APRF1 functions like Swd2 (yeast)/WDR82 (metazoan) in a CPF-like phosphatase module. Interestingly, in all species studied, the same components are involved in 3′ end processing and transcription termination, but they have different affinities. In yeast, the CPF complex contains all enzymatic activities required for mRNA 3′ end processing and transcription termination (cleavage, polyadenylation, and dephosphorylation). In humans, the phosphatases are present within the activated 3′ end-processing machinery, pulled down on an RNA substrate,^66^ but are not constitutive components of CPSF. It remains unclear whether the phosphatase module in plants is constitutively associated with the cleavage and polyadenylation machinery, but our mass spectrometry data suggest that it may be a regulated interaction, similar to the human situation.^67^ The robust interaction between APRF1 and the CTD phosphatase CPL3, which has been shown to be able to dephosphorylate Ser2, Ser5, and Ser7,^68^ may reflect either a simplified termination machinery in plants or the existence of specialized sub-complexes working in an environmentally/developmentally regulated manner.

Another interesting evolutionary difference is the clear sub-functionalization of the *Arabidopsis* Swd2 homologs *APRF1* and *S2LB*.^69^ In yeast, Swd2 is a single-copy gene whose product is found in two protein complexes with antagonistic functions: COMPASS^25^ and mRNA 3′ end-processing machinery.^26,70^ *Arabidopsis* orthologs do not play overlapping roles in these two complexes. S2LB interacts *in vivo* with the methyltransferase SDG2 and the structural component of COMPASS WDR5, controlling H3K4me3 levels genome-wide at thousands of gene promoters.^24^ In contrast, APRF1 interacts with LD, other components of the FLD complex, and TOPP4. This functional divergence is confirmed through analysis of mutants affecting *FLC* expression. Like *atx1-2* and other mutants in the *Arabidopsis* COMPASS machinery,^29^ *s2lb* mutants reduce *FLC* and *COOLAIR* expression due to their impaired ability to deposit H3K4me3 at the locus.

The mechanism we describe links transcription termination to histone demethylase activity, resulting in graded repression of subsequent transcription. Our accompanying paper then takes this mechanism and describes how it promotes the switch to Polycomb silencing.^18^ Only by combining the extensive genetics and proteomic analysis described in this paper with the modeling/experimental validation work described in the accompanying paper could we fully describe the whole mechanism. A low transcriptional state influenced by proximal termination and H3K4 demethylation reduces the antagonism to Polycomb silencing and leads to a stable PRC2 epigenetic switch, with sufficient feedback to maintain the silenced state through DNA replication and cell division. We have generated an animation (Video S1) to help explain these molecular feed-backs and how this mechanism leads to the stable expression of *FLC* in one of two stable expression states. These transcription-termination/chromatin-silencing mechanisms have proven difficult to dissect at the molecular level in many systems but are likely to be the basis of many epigenetic switches. There are parallels with the mechanism described here and those of the CPF-triggered heterochromatin silencing in *S. pombe*.^71^ 3′ processing has been extensively linked to chromatin silencing in *S. pombe*^72,73^ and found to be important for plants to cope with heat shock.^74^ In *S. cerevisiae*, a direct connection between a lysine demethylase KDM5 and the CPF was reported.^75^ In human cells, a clear genome-wide correlation has been found between human FLD and APRF1 homologs (LSD1 and WDR82) and Pol II,^59,76^ and there is a direct relationship between LSD1 and an RNA helicase involved in R-loop resolution and PRC2-mediated silencing.^77^ Continued mechanistic dissection is therefore likely to elaborate generally important concepts in chromatin silencing.

### Limitations of the study

The work focuses on the regulation of one plant developmental regulator to discover a link between transcriptional termination and chromatin remodeling. This could be a general mechanism for establishment of chromatin silencing. Future work will be required to define how generic this mechanism is and whether or not other targets share molecular features with *FLC*.

We were able to show that APRF1-LD-TOPP4 form a CPF phosphatase sub-module, but we do not know whether other CPF phosphatase components are associated with this complex, as in yeast, or whether they are more loosely associated, as in mammals. There is the possibility of sub-complex specialization in particular environmental/developmental contexts.

Finally, the substrate of the CPF phosphatase module remains to be established. Work in yeast and mammals has suggested that both RNA Pol II CTD and/or elongating factors such as SPT5 may be de-phosphorylated by the complex. Our results are compatible with both scenarios and thus have not helped to differentiate between these possibilities.

## Star★Methods

Detailed methods are provided in the online version of this paper and include the following:

● KEY RESOURCES TABLE
● RESOURCE AVAILABILITY
  ○ Lead contact
  ○ Materials availability
  ○ Data and code availability
● EXPERIMENTAL MODEL AND STUDY PARTICIPANT DETAILS
● METHOD DETAILS
  ○ Gene expression analyses
  ○ ChIP
  ○ CrossLinked Nuclear ImmunoPrecipitation and Mass Spectrometry (CLNIP-MS)
  ○ Protein co-immunoprecipitation in *Nicotiana benthamiana*
  ○ Preparation of Chromatin-bound RNA
  ○ Quant-seq
  ○ plaNET-seq
  ○ AlphaFold2 protein interaction prediction
  ○ Bioinformatic analyses
● QUANTIFICATION AND STATISTICAL ANALYSIS

## Star★Methods

### Key Resources Table

**Table:** 

REAGENT or RESOURCE	SOURCE	IDENTIFIER
Antibodies		
Anti-H3 antibody	Abcam	Cat: ab176842; RRID:AB_2493104
Anti-H3K27me3 antibody	Abcam	Cat: ab192985; RRID:AB_2650559
Anti-H3K36me3 antibody	Abcam	Cat: ab9050;RRID:AB_306966
Anti-H3K4me1 antibody	Abcam	Cat: ab8895; RRID:AB_306847
Anti-FLAG ® antibody	Merck	Cat: F1804; RRID:AB_262044
Anti-GFP antibody	Abcam	Cat: ab290; RRID:AB_303395
Anti-Tyr1P antibody	Merck	Cat: MABE350
Anti-Ser2P antibody	Diagenode	Cat: C15200005-50; RRID:AB_2713925
Anti-Ser5P antibody	Diagenode	Cat: C15200007-50; RRID:AB_2713926
Anti-FLAG ® M2 HRP	Merck	Cat: A8592; RRID:AB_439702
Anti-GFP	Santa Cruz	Cat: sC9996HRP; RRID:AB_627695
Bacterial and virus strains		
*Escherichia coli* HST08	Takara	Cat: 636763
*Agrobacterium tumefaciens* GV3101 competent cells	Lab stock	N/A
*Agrobacterium tumefaciens* C58C1 competent cells	Lab stock	N/A
Chemical, peptides, and recombinant proteins		
Anti-FLAG ® Affinity Gel	Merck	Cat: A2220
A*ar*I	ThermoFisher Scientifc	Cat: ER1581
rAPid	Merck	Cat: 4898133001
T4 Polynucleotide Kinase	New England BioLabs	Cat: M0201S
T4 Ligase	New England BioLabs	Cat: M0202S
Turbo DNase	Ambion	Cat: AM1907
SuperScript IV	Invitrogen	Cat: 18090050
Phenol solution saturated with 0.1 M Citrate	Merk Life Science UK Ltd	Cat: P4682
RNaseOUT RNase Inhibitor	ThermoFisher Scientifc	Cat: 10777019
Lightcycler 480 Sybr Green I	Roche Diagnostics Ltd	Cat: 04887352001
InFusion kit	Takara	Cat: 638945
cOmplete protease inhibitors	Merck	Cat: 11697498001
Protein A-coated Dynabeads	ThermoFisher Scientifc	Cat: 10001D
M-270 Epoxy Dynabeads	ThermoFisher Scientifc	Cat: 14311D
EGS (etilenglicol bis(succinimidil succinato))	ThermoFisher Scientifc	Cat: 21565
PhosSTOP	Merck	Cat: 4906845001
Chelex resin	BioRad	Cat: 1421253
Proteinase K	Merck	Cat: 3115887001
PSMF (Phenylmethanesulfonyl fluoride)	Roche Diagnostics Ltd	Cat: 10837091001
Percoll	Merck	Cat: P7828
Benzonase	Merck	Cat: 70746
SuperSignal West Pico	ThermoFisher Scientifc	Cat: 34580
SuperSignal West Femto	ThermoFisher Scientifc	Cat: 34095
PageRuler™ Prestained Protein Ladder	ThermoFisher Scientifc	Cat: 26616
TRIzol	Invitrogen	Cat: 15596026
Phenol:Chlorophorm:isoamylalcohol	Sigma	Cat: P3803-100ML
RNA glycoblue	ThermoFisher Scientifc	Cat: AM9516
RNasin	Promega	Cat: N2515
DNase I	Roche Diagnostics Ltd	Cat: 03724778103
Critical commercial assays		
RNeasy miniprep kit	Qiagen	Cat: 74106
Qubit dsDNA HS assay	ThermoFisher Scientifc	Cat: Q32851
*mybaits*	ArborBiosciences	N/A
Directzol	Zymo Research	Cat: R2063
NEXTflex Small RNA-seq kit v3	PerkinElmer	Cat: 5132-05
RNAclean XP beads	Beckman Coulter	Cat: A63987
Deposited data		
APRF1-3xFLAG proteomics	PRIDE	PXD049114
Quant-seq *ColFRI* vs *aprf1-9 FRI*	SRA	PRJNA978558
Quant-seq Col-0 vs *fld-4*	SRA	PRJNA1076161
plaNET-seq ColFRI vs *aprf1-9 FRI*	SRA	PRJNA1076151
Experimental models: Organisms/strains		
*Arabidopsis thaliana* Col-0	Standard accession	N/A
Col*FRI*	Standard accession	N/A
*fca-9*	Fang et al.^10^	N/A
*fld-4*	Fang et al.^10^	N/A
*ld-1*	Fang et al.^10^	N/A
*aprf1-9*	Arabidopsis Stock Centre	N858279
*s2lb*	Fiorucci et al.^24^	N/A
*aprf1-9 s2lb*	Fiorucci et al.^24^	N/A
*atx1-2*	Pien et al.^78^	N/A
*fld-4; FLD_pro_:3xFLAG-FLD; fri*	Inagaki et al.^12^	N/A
*fld-4; FLD_pro_:3xFLAG-FLD; FRI*	Inagaki et al.^12^	N/A
*APRF1_pro_:APRF1 -3xFLAG*	Qi et al.^22^	N/A
*aprf1-10*	This paper	N/A
*APRF1_pro_:APRF1 -mVENUS*	This paper	N/A
*TOPP4_pro_:TOPP4-3xFLAG*	This paper	N/A
*TCP14_pro_:TCP14-FLAG*	Weßling et al.^79^	N/A
*aprf1-9FRI*	This paper	N/A
*aprf1-9 fca-9*	This paper	N/A
*aprf1-9 fld-4*	This paper	N/A
*aprf1-9 ld-1*	This paper	N/A
Oligonucleotides		
Primers used in this study are listed in Table S4.	This paper	N/A
Recombinant DNA		
Plasmid: pKI1.1R	Addgene	Cat: 85808
Plasmid: pKI1.1R + sgRNA-APRF1	This paper	N/A
Plasmid: pCAMBIA1300 + *APRF1_pro_:APRF1-mVENUS*	This paper	N/A
Plasmid: pCAMBIA1300 + *TOPP4_pro_:TOPP4-3xFLAG*	This paper	N/A
Software and algorithms		
Proteome Discoverer 3.1	ThermoFisher Scientifc	https://www.thermofisher.com
Microsoft Excell	Microsoft	https://www.microsoft.com
GraphPad Prism 10	GraphPad	https://www.graphpad.com
FastQC v0.11.7	N/A	https://github.com/s-andrews/FastQC
Cutadapt 1.18	Martin^80^	https://github.com/marcelm/cutadapt
STAR v2.6.1a	Dobin et al.^81^	https://github.com/alexdobin/STAR
UMI-tools v1.1.1	Smith et al.^82^	https://github.com/CGATOxford/UMI-tools/releases
Trimmomatic V0.39	Bolger et al.^83^	https://github.com/usadellab/Trimmomatic

## Resource Availability

### Lead contact

Further information and requests for resources and reagents should be directed to and will be fulfilled by the lead contact, Caroline Dean (caroline.dean@jic.ac.uk).

### Materials availability

The plasmids and transgenic plants generated in this study are available from the lead contact upon request.

### Experimental Model and Study Participant Details

*Arabidopsis thaliana* plants were used in this study. All mutants and transgenics were in Columbia (Col) background and are homozygous for the indicated genotype. The *aprf1-9* mutant seeds (WiscDsLox489-492K11, N858279) were obtained from NASC (Not-tingham Arabidopsis Stock Centre). The *s2lb* mutant and the *aprf1-9 s2lb* double mutant, previously described in Fiorucci et al.,^24^ were kindly provided by Fredy Barneche. The *atx1-2* was previously described in Pien et al.^78^ Transgenics *fld-4*; *FLD_pro_:3xFLAG-FLD* and *APRF1_pro_:APRF1-3xFLAG* were previously described in Inagaki et al.,^12^ Qi et al.,^22^ and were shared by Soichi Inagaki and Xin-Jian He, respectively.

The *aprf1-10* mutant harbours a 5-nt deletion in *APRF1*. The *aprf1-10* deletion creates a new target for the M*ae*II (target ACGT) restriction enzyme. To generate the *aprf1-10* we employed the CRISPR/Cas9 plasmid pKI1.1R following the protocol described.^84^ Briefly, pKI1.1R plasmid (Addgene #85808) was linearized by incubating 1.5 μg of the plasmid with A*ar*I restriction enzyme for 16 h, and then dephosphorylated using the alkaline phosphatase rAPid (Roche). A target-specific gRNA was designed using CRIPR-P 2.0 (http://crispr.hzau.edu.cn/CRISPR2). Oligonucleotides harbouring the gRNA target (sgRNA_APRF1_F and sgRNA_APRF1_R; Table S4) were hybridised by slow cooling down from 95-25°C and then phosphorylated using the T4 Polynucleotide Kinase (NEB). The digested plasmid and the hybridised oligonucleotides were ligated using the T4 ligase (NEB) and then transformed in *Escherichia coli* HST08 competent cells (Takara). The sequence integrity of inserts carried by transformants were verified by Sanger sequencing. The plasmid was then transfer to *Agrobacterium tumefaciens* C58C1 strain by electroporation. T_1_ plants carrying the construct were selected on MS media supplemented with 15 μg/ml of Hygromycin. Next generation plants were counter-selected to find transgenefree individuals carrying the homozygous mutation.

Seeds were surface sterilized in 40 % v/v commercial bleach for 10 min and rinsed 4 times with sterile distilled water. Seeds were then sown on standard half-strength Murashige and Skoog (MS) medium (0.22% MS, 8% plant agar) media plates and kept at 4°C in darkness for 3 days before being transferred to long day photoperiod conditions (16 h of light, 8 h dark). All RNA and protein experiments were done using 14-days old seedlings unless otherwise specified.

## Method Details

### Gene expression analyses

Seedlings were harvested, and RNA was extracted with the hot phenol method as previously described.^85,86^ TURBO DNase (Ambion) was used to remove genomic DNA contamination before reverse transcription. cDNA was synthesized using SuperScript IV (Invitrogen) and gene-specific primers (Table S4). qPCR analyses were performed, and data was normalized to the indicated housekeeping gene or genes.

### ChIP

2.5 gr of seedlings were crosslinked with 1% formaldehyde in 1X PBS for 12 min by vacuum infiltration, followed by addition of glycine (final concentration 125 mM) with another 7 min of vacuum infiltration. Tissue was then ground to fine powder with liquid nitrogen. Ground tissue was resuspended in 35 μL of Honda Buffer (20 mM Hepes, 0.44 M sucrose, 1.25 % Ficoll, 2.5% Dextran, 10 mM MgCl_2_, 0.5% Triton X-100, 5 mM DTT, 1x Roche protease inhibitor mixture), filtered through two layers of Miracloth, and centrifuged at 2500 xg for 15 min. Nuclei pellet was then washed once more with 1.6 μL of Honda Buffer.

For histone ChIP, nuclear pellets were resuspended in Nuclei Lysis Buffer (50 mM Tris-HCl pH 8, 10 mM EDTA, 1 % SDS), and sonicated 4 x 5 min (30 sec ON/ 30 sec OFF) using a Diagenode Bioruptor on Medium setting. IP was performed by incubating 140 μl of sonicated chromatin diluted ten times with ChIP dilution buffer (16.7 mM Tris-HCl pH 8, 1.2 mM EDTA, 1.1 % Triton X-100, 167 mM NaCl, 1X cOmplete protease inhibitors) with 15 μl of Protein A-coated Dynabeads (Invitrogen) previously incubated for 2 h with either 2.5 μg of anti-H3 (ab176842), anti-H3K27me3 (ab192985), anti-H3K36me3 (ab9050), or H3K4me1 (ab8895) and incubated overnight at 4°C on a rotator wheel.

For FLAG-FLD and GFP-LD ChIP, nuclei were obtained as described above, but for FLAG-FLD the crosslinking buffer was supplemented with 1.5 mM of EGS (ethylene glycol bis(succinimidyl succinate); ThermoFisher). Nuclear pellets were suspended in RIPA buffer (50 mM Tris-HCl, 150 mM NaCl, 1% Nonidet P-40, 0.5% NaDeoxycholate, 0.1% SDS, 1x Roche protease inhibitor mixture) and sonicated 5 times x 5 min (30 s ON/ 30 s OFF) with the Bioruptor in high setting. Undiluted chromatin was incubated overnight at 4°C with either 1.5 μg Dynabeads M-270 Epoxy preincubated with 1.5 μl anti-FLAG (Anti-FLAG® M2 / F1804, Merck) for FLAG-FLD or 15 μl Protein-A coated Dynabeads with 2.5 μl anti-GFP (ab290, Abcam), for GFP-LD.

Beads were then washed twice with Low Salt Wash Buffer (150 mM NaCl, 0.1 % SDS, 1% Triton X-100, 2 mM EDTA, 20 mM Tris-HCl pH 8, 1x Roche protease inhibitor mixture), twice with High Salt Wash Buffer (500 mM NaCl, 0.1 % SDS, 1 % Triton X-100, 2 mM EDTA, 20 mM Tris-HCl 8, 1x Roche protease inhibitor mixture), and twice with TE wash buffer (10 mM Tris-HCl pH 8, 1 mM EDTA, 1x Roche protease inhibitor mixture).

For RNA Pol II ChIP, nuclei were obtained as described for histone ChIP, complementing the Honda Buffer with 1x of PhosSTOP (Merck). Nuclear pellet was suspended in 1 μl of TAP buffer (100 mM NaCl, 20 mM Tris-HCl pH 8, 2.5 mM EDTA, 10 % glycerol, 1 % Triton, 1x of PhosSTOP, 1x Roche protease inhibitor mixture) and given 20 strokes with the Dounce Homogenizer. The resulting solution was sonicated 4 x 10 min (15 s ON/ 45 s OFF) with the Bioruptor in low setting. 250 μl of undiluted chromatin was incubated overnight at 4°C with Dynabeads M-270 Epoxy preincubated with anti-Tyr1P (MABE350, Merck), anti-Ser2P (C15200005-50, Diagenode), or anti-Ser5P (C15200007-50, Diagenode). Then beads were washed twice for 15 min with Low Salt and High Salt buffers like for histones but including 1x of PhosSTOP. Then beads were washed for 15 min with the LiCl buffer (250 mM LiCl, 0.5 % NP40, 2.5 mM EDTA, 0.05 % NaDeoxycholate, 20 mM Tris-HCl pH 8, 1x of PhosSTOP, 1x Roche protease inhibitor mixture), and the TE buffer (same as for histones plus 1x PhosSTOP). In all cases, after IP, DNA was then eluted and reverse-crosslinked by incubating the beads at 95°C for 10 min in presence of 100 μl of 10 % Chelex resin (BioRad), treated with Proteinase K (Roche) for 1 h at 45°C, and incubated again at 95°C for 10 min to inactivate the Proteinase K. Finally, DNA was purified using the ChIP DNA Clean & Concentrator kit (Zymo Research).

### CrossLinked Nuclear ImmunoPrecipitation and Mass Spectrometry (CLNIP-MS)

10-days-old *APRF1_pro_:APRF1-3xFLAG*^22^ and Col-0 (control) seedlings were crosslinked with 1% formaldehyde in 1X PBS for 10 min. Three biological replicates for each genotype were used. 2 g of tissue per biological replicate was ground to a fine powder and resuspend in 30 μL of Honda Buffer, supplemented with 1 mM phenylmethylsulfonyl fluoride (PMSF). The suspension was filtered through a double layer of Miracloth and centrifuged at 2,000 g for 15 min at 4°C. The nuclei pellet was washed once in 5 μl of Honda buffer then purified on a Percoll density gradient as follows: 2 μl of 75% Percoll (Merck, P7828) in Honda buffer topped with 2 μl of 40% Percoll in Honda buffer topped with the nuclei pellet resuspended in Honda buffer in a 15 μl tube. Purified nuclei were obtained in between the layers containing 40% and 75% Percoll after centrifuging at 7,000 g for 30 min at 4°C and washed once more in 6 μl Honda buffer. The nuclei pellet was resuspended in 350 μl of Benzonase buffer (50 mM Tris pH 8.0, 1 mM MgCl2, and 1X cOmplete protease inhibitors), and incubated with 1 μl of Benzonase (Millipore, 70746) for 40 min at 4°C. Nuclei were then incubated for 30 min at 4°C after adding 1% SDS, then diluted with ChIP dilution buffer to a concentration of 0.5% SDS in the samples, then sonicated using the Bioruptor in Medium setting for 3 cycles of 5 min (30 sec on/ 30 sec off). Samples were centrifuged at 10,000 g for 1 min, and the supernatant was diluted with ChIP dilution buffer to a concentration of 0.1% SDS in the sample. IP was performed overnight at 4°C after adding the antibody-beads complex. 1.5 mg of anti-FLAG (Sigma, F1804) antibody was coupled to 1.5 mg of M-270 epoxy Dynabeads (Invitrogen, 14311D) following the manufacturer’s procedure and used per IP reaction. After IP, samples were washed with 1 μl of IP wash buffer (50 mM Tris pH 8.0, 150 mM NaCl, 1% Triton X-100, and 0.5% IGEPAL CA-630) 4 times for 5 min each, and then resuspend in 50 μl of SDS buffer (20 mM Tris pH 8.0 and 2% SDS), and heated to 90°C for 15 min. Then, the samples were separated from the beads and proteins precipitated based on Pankow et al.^87^ by adding 1.1/4.4 chloroform/methanol mix. The protein pellet was then washed twice with methanol, once with acetone, and air dried.

For Mass Spectrometry, protein pellets were resuspended in 50 μl of 1.5% sodium deoxycholate (SDC; Merck) in 0.2 M EPPS-buffer (Merck), pH 8.5 and vortexed under heating. Cysteine residues were reduced with dithiothreitol, alkylated with iodoacetamide, and the proteins digested with trypsin in the SDC buffer according to standard procedures. After the digest, the SDC was precipitated by adjusting to 0.2% trifluoroacetic acid (TFA), and the clear supernatant subjected to C18 SPE using home-made stage tips with C18 Reprosil_pur 120, 5 mm. Aliquots were analysed by nanoLC-MS/MS on an Orbitrap Eclipse™ Tribrid™ mass spectrometer coupled to an UltiMate® 3000 RSLCnano LC system (Thermo Fisher Scientific, Hemel Hempstead, UK). The samples were loaded onto a trap cartridge (PepMap™ Neo Trap Cartridge, C18, 5um, 0.3x5mm, Thermo) with 0.1% TFA at 15 μl min-1 for 3 min. The trap column was then switched in-line with the analytical column (Aurora Frontier TS, 60 cm nanoflow UHPLC column, ID 75 mm, reversed phase C18, 1.7 mm, 120 A° ; IonOpticks, Fitzroy, Australia) for separation at 55°C using the following gradient of solvents A (water, 0.1% formic acid) and B (80% acetonitrile, 0.1% formic acid) at a flow rate of 0.26 μl min-1: 0-3 min 1% B (parallel to trapping); 3-10 min increase B (curve 4) to 8%; 10-102 min linear increase B to 48; followed by a ramp to 99% B and re-equilibration to 0% B, for a total of 140 min runtime. Mass spectrometry data were acquired with the FAIMS device set to three compensation voltages (-35V, -50V, -65V) at standard resolution for 1.0 s each with the following MS settings in positive ion mode: OT resolution 120 K, profile mode, mass range m/z 300-1600, normalized AGC target 100%, max inject time 50 ms; MS2 in IT Turbo mode: quadrupole isolation window 1 Da, charge states 2-5, threshold 1e4, HCD CE = 30, AGC target standard, max. injection time dynamic, dynamic exclusion 1 count for 15 s with mass tolerance of ±10 ppm, one charge state per precursor only.

The mass spectrometry raw data were processed and quantified in Proteome Discoverer 3.1 (ThermoFisher) using the search engine CHIMERYS (MSAID, Munich, Germany); all mentioned tools of the following workflow are nodes of the proprietary Proteome Discoverer (PD) software. The Arabidopsis TAIR10 protein database (https://arabidopsis.org; 32785 entries) was imported into PD adding a reversed sequence database for decoy searches; in the same way, a small customs database with the APRF1-FLAG protein sequence and a database for common contaminants (https://maxquant.org, 245 entries) was also included. The CHIMERYS database search was performed with the inferys_3.0.0_fragmentation prediction model, a fragment tolerance of 0.3 Da, enzyme trypsin with 2 missed cleavages, variable modification oxidation (M), fixed modification carbamidomethyl (C) and FDR targets 0.01 (strict) and 0.05 (relaxed). The workflow included the Minora Feature Detector with min. trace length 5, S/N 2.5, PSM confidence high. The consensus workflow in the PD software was used to evaluate the peptide identifications and to measure the abundances of the peptides based on the LC-peak intensities. For identification, an FDR of 0.01 was used as strict threshold, and 0.05 as relaxed threshold.

For quantification, three replicates of *APRF1_pro_:APRF1-FLAG* and Col-0 were measured. In PD3.1, the following parameters were used for ratio calculation: normalisation on total peptide abundances, protein abundance-based ratio calculation using the top three most abundant peptides, missing values imputation by low abundance resampling, hypothesis testing by t-test (background based), adjusted p-value calculation by BH-method. The results were exported into a Microsoft Excel table including data for protein abundances, ratios, p-values, number of peptides, protein coverage, the CHIMERYS identification score and other important values. The mass spectrometry proteomics data have been deposited to the ProteomeXchange Consortium via the PRIDE^88^ partner repository with the dataset identifier PXD049114 and 10.6019/PXD049114.

### Protein co-immunoprecipitation in *Nicotiana benthamiana*

To generate *APRF1_pro_:APRF1-mVENUS* and *TOPP4_pro_:TOPP4-3xFLAG* the genomic region of both genes including a region of around 1.5 kb upstream their transcription start sites were amplified by PCR and cloned by the InFusion cloning (Takara) into the pCAMBIA1300 in frame with the coding sequences of mVENUS or the 3xFLAG peptide, respectively, using the primers listed in Table S4. After verification by sequencing, both transgenes were transferred to the strain GV3101 of *Agrobacterium tumefaciens*. As a negative control, we used FLAG-tagged version of the Arabidopsis TCP14, a transcription factor involved in plant immunity,^79^ kindly shared by Jonathan D. G. Jones (The Sainsbury Laboratory). Before co-infiltration, protein levels of individual proteins were verified by single agroinfiltrations. Overnight grown bacteria were used to agroinfiltrate adjusting the OD600 to their protein levels. Cells were resuspended in the Infiltration Buffer (10 mM MES pH 5.6, 10 mM MgCl2, 1 mM acetosyringone) and used to agroinfiltrated *N. benthamiana* leaves. Co-infiltrated leaves with either APRF1-mVENUS + TOPP4-3xFLAG or APRF1-mVENUS + TCP14-FLAG were grown 2 more days before collecting the material. Around 0.85 gr of infiltrated tissue was harvested and ground to a fine powder with liquid nitrogen and homogenized in ice-cold extraction buffer (10 % glycerol, 25 mM Tris-HCl pH 7.5, 1 mM EDTA, 150 mM NaCl, 2 % PVP, and 0.2 % Tween-20). The lysate was homogenized, washed twice, and filtered through Miracloth. The supernatant was incubated with 30 μl of washed anti-FLAG M2 Affinity Gel (A2220, Millipore) for 2 hours at 4°C in rotation. The beds were washed four times with IP wash buffer (25 mM Tris-HCl pH 7.5, 1 mM EDTA, 150 mM NaCl, 0.2 % Tween-20, 1 mM DTT, 1x cOomplete protease inhibitors) at 4°C, and resuspended in SDS-loading buffer with 10 mM DTT. Proteins were released and denatured after incubation at 95°C for 7 min and resolved by SDS-PAGE. Anti-FLAG M2-HRP (A8592, Sigma), or anti-GFP (sC9996HRP, Santa Cruz) antibodies were used for Western blot to detect TOPP4-FLAG (expected size: 39 kDa), TCP14-FLAG (52 kDa), or APRF1-mVENUS (62 kDa), respectively. The chemiluminescence substrate SuperSignal West Pico (34580) was used for FLAG immunoblots and SuperSignal West Femto (34094, Thermo) for GFP blots. Uncropped blots are shown in Figure S8.

### Preparation of Chromatin-bound RNA

Chromatin-bound RNA was isolated as previously described.^11^ Nuclei from 2-2.5 gr of non-crosslinked seedlings were obtained with Honda Buffer, supplemented with 20 U/mL RNase inhibitor RNase Out (Invitrogen), 1 mM PMSF, and 50 ng/mL of yeast tRNA. Nuclear pellet was rinsed with 500 μl of resuspension buffer (20 mM Tris pH 8, 75 mM NaCl, 0.5 mM EDTA, 1 mM DTT, 0.125 mM PMSF, 50 % glycerol, 1x Roche complete, 20 U/mL RNase Out) and centrifuged at 4000 xg at 4°C for 3 min. Nuclei pellet was weighed and resuspended in an equal volume of Resuspension Buffer. The suspension was then washed with two volumes of Urea Wash Buffer (20 mM Tris pH8, 300 mM NaCl, 7.5 mM MgCl_2_, 0.25 mM EDTA, 1 mM DTT, 1 M Urea, 1 % NP-40, 1x Roche complete, 20 U/mL RNase Out), pipetting up and down 30 times, and spun at 8,000 xg for 1 min at 4°C. Nuclei pellet was resuspended again with 1 volume of Resuspension buffer and washed with 1 volume of Urea Wash Buffer, pipetting 30 times up and down, and spun for 1 min at 8,000 xg and 4°C. Finally, nuclear pellet was dissolved in 1 μl of TRIzol (Invitrogen), adding 0.2 μl of chloroform and shaking vigorously by hand for 15 s. Then the suspension was incubated at RT for 2 min and centrifuged at 12,000 xg for 15 min at 4°C. The aqueous phase was taken and mixed with an equivalent volume of Phenol:Chloroform:Isoamyl alcohol (25:24:1, Sigma), shaken for 10 min at room temperature, and centrifuged at RT and 12,000 xg for 10 min. The aqueous phase was then transferred to another tube and followed two precipitations, first with isopropanol, sodium acetate, and RNA GlycoBlue and another one with LiCl. Finally, RNA was dissolved and treated with DNase Turbo (Ambion) and used as template for reverse transcription with gene-specific primers (Table S4).

### Quant-seq

For Quant-seq experiments, total RNA was isolated as for qPCR and further cleaned up using the Qiagen RNeasy miniprep kit (74106). Library preparation, sequencing, and data analysis were carried out by Lexogen GmbH (Austria). Sequencing-ready libraries were generated from 100 ng of input RNA using a QuantSeq 3’ mRNA-Seq Library Prep Kit REV for Illumina (015UG009V0271) following standard procedures. RNA integrity, and Indexed libraries quality were assessed on a Fragment Analyzer device (Agilent Technologies) using a DNF-471 RNA Kit and HS-DNA assay, respectively. Libraries were quantified using a Qubit dsDNA HS assay (Thermo Fisher). A sequencing-ready pool of indexed libraries were sequenced on an Illumina NextSeq 2000 with a 100-cycle cartridge using the Custom Sequencing Primer (CSP). FastQC version v0.11.7 was used to verify the read quality and cutadapt version 1.18^80^ for read adapter trimming. Clean reads were mapped to the latest version of the Arabidopsis genome (TAIR10) with a spliceaware aligner STAR version 2.6.1a.^81^ Differentially expressed genes (DEGs) between Col*FRI* and *aprf1-9 FRI* and Col-0 and *fld-4* are listed in Tables S2 and S3, respectively.

For enrichment of *FLC* and selected control genes, 4,861 synthetic 80-nt biotinylated RNA probes were synthesized, complementary to 32 padded gene sequences at 2x bp tiling density (±1kb padding) (*mybaits*; ArborBiosciences; Data S1). Selected libraries were pooled equimolar and in-solution target capture was carried out using the manufacturers standard sensitivity protocol with a bait annealing temperature of 65°C. The bait enriched library pool was sequenced on an Illumina NextSeq 2000 with a 100-cycle cartridge using the Custom Sequencing Primer (CSP). Raw reads have been deposited on Short Read Archive (SRA) under the references PRJNA978558 and PRJNA1076161.

### plaNET-seq

Nascent transcript isolation was adapted from Kindgren et al.^51^ 3 gr of Arabidopsis seedlings were flash frozen in liquid nitrogen and extracted with NUC1 (0.4 M sucrose, 10 mM Tris–HCl pH 8.0, 10 mM MgCl_2_,5 mM β-mercaptoethanol, proteinase inhibitor [Complete; Roche], phosphatase inhibitor [PhosSTOP; Roche] and RNase inhibitor [RNasin; Promega]). Once homogeneous, samples were centrifuged at 5000 g for 20 minutes and the pellet was washed with 1 μl NUC2 buffer (0.25 M sucrose, 10 mM Tris–HCl pH 8.0, 10 mM MgCl_2_, 5 mM β-mercaptoethanol, proteinase inhibitor, phosphatase inhibitor, RNase inhibitor and 0.3 % Tween-20). Nuclei were suspended in 0.3 μl NUC3 buffer (1.7 M sucrose, 10 mM Tris–HCl pH 8.0, 2 mM MgCl_2_,5 mM β-mercaptoethanol, proteinase inhibitor tablet, phosphatase inhibitor, RNase inhibitor (Recombinant RNasin 20 U/ml; Promega) and 0.15 % Tween-20) and carefully layered over 0.9 μl of NUC3 in prechilled microcentrifuge tubes before centrifugation at 16000 g for 60 min at 4°C. Purified nuclei were lysed in 1.5 μl plaNET-seq lysis buffer (0.3 M NaCl, 20 mM Tris–HCl pH 7.5, 5 mM MgCl_2_, 5 mM DTT, proteinase inhibitor, phosphatase inhibitor, RNase inhibitor, 0.5% Tween-20 and DNaseI [400 U/ml; Roche]) at 4°C shaking at 2000 rpm. Lysate was centrifuged at 10000 g at 4°C for 10 minutes and supernatant was transferred to Dynabeads M-270 (Invitrogen) coupled to either CTD Ser2P (C15200005; Diagenode) or Ser5P (C15200007; Diagenode) antibodies. After 2 hours incubation at 4°C, immunocomplexes were washed gently six times with wash buffer (0.3 M NaCl, 20 mM Tris-HCl pH 7.5, 5 mM MgCl_2_, 5 mM DTT, proteinase in-hibitor RNase inhibitor and phosphatase inhibitor) and dissolved in 1 μl TRIzol (Invitrogen), followed by isolation of the nascent RNA with on column DNA digestion (RNA microprep kit; Direct-zol).

For plaNET-seq library construction, 100 ng of nascent RNA was used as input to construct plaNETseq libraries using NEXTflex Small RNA-seq kit v3 (PerkinElmer) with a modified protocol. After 3’ adapter ligation, RNA was fragmented by incubation with alkaline solution (100 mM NaCO3 pH 9.2, 2 mM EDTA) at 95°C for 5 minutes (Churchman & Weissman 2012), followed by clean up (RNA-clean XP beads; Beckman Coulter), PNK treatment (NEB) for 20 min at 37°C and reannealing of the RT-primer (8 mM). Library con-struction continued from the adapter inactivation step of the manufacturer’s protocol. Libraries were quantified using a Qubit dsDNA HS assay (Thermo Fisher). A sequencing-ready pool of indexed libraries were sequenced on an Illumina Xten PE150 at Beijing Genomics Institute. Raw reads have been deposited on SRA under the reference PRJNA1076151.

For plaNET-seq data analysis, Unique Molecular Identifiers (UMIs) were first trimmed from the read and appended to the read name with UMI-tools v1.1.1,^82^ followed by adapter and read quality trimming with trimmomatic v0.39.^83^ R2 reads were mapped to the Arabidopsis genome (TAIR10) with a splice-aware aligner STAR version 2.7.10a.^81^ PCR duplicates were filtered from the alignment files with UMI-tools, low mapping quality reads were removed (MAPQ>10 samtools v1.9) and reads were flipped to restore the original RNA read strand orientation. Read 3’ends that overlap with 5’ and 3’ splice sites (and likely represent co-transcriptional splicing intermediates) were removed before generating strand specific coverage files for visualisation of nascent transcripts. For generate the metaplots, gene models from Araport 11 were used to define the TSS and the TTS as described in Kindgren et al.^51^ The gene list was filtered to remove genes overlapping features within 500 bp of the TSS or TTS. For the remaining genes average signal for each position was calculated around the TSS or TTS (±500 bp) and divided into 5 bp bins. For each position 0.01% of extreme values were trimmed before averaging. The mean coverage for each genotype was plot for the given genomic interval with the shaded area indicating the 95% confidence interval for the mean. For the Readthrough analyses, the total binned signal between 150-450 bp downstream of the TTS was calculated for each sample to generate the readthrough signal. The gene 3’end signal was calculated by taking the total signal from a random but equal number of bins within the 3’end 1kb-0.1kb upstream of the TTS. Readthrough rate was expressed as a ratio of readthrough signal relative to 3’ end signal for each replicate. One-way ANOVA was performed to determine statistical significance of the differences between genotypes.

### AlphaFold2 protein interaction prediction

AlphaFold models were predicted using the Colab notebook running a slightly simplified version of AlphaFold v2.3.2.^44^ For the Arabidopsis LD–APRF1 complex LD aa566-661 and APRF1 aa1-330 were used. For the human PNUTS–WDR82 complex PNUTS aa380-530 and WDR82 aa1-313, and for the yeast Ref2–Swd2 complex Ref2 aa406-533 and Swd2 aa1-329 were used. The number of recycles were set to 3 and models included a final relaxation stage. PAE plots indicating the quality of the prediction and LDDT plots are shown in Figure S15. Final models were imported into and colour figures were prepared with PyMOL (v2.1, Schrödinger).

### Bioinformatic analyses

To design the sgRNA to edit *APRF1* via CRISPR/Cas9 we used the CRISPR-P 2.0 (http://crispr.hzau.edu.cn/cgi-bin/CRISPR2/CRISPR), selecting the canonical NGG PAM motif, and the Arabidopsis TAIR10 as a target genome. Protein alignments were performed with MEGA X^89^ using the MUSCLE^90^ algorithm with default parameters, and shaded with the “Colour Align Conservation” tool from the Sequence Manipulation Suite.^91^ To find putative PNUTS homologs in Arabidopsis, BLASTP,^92^ PSI-BLAST, and DELTA-BLAST^93^ searches were performed at NCBI site using an alignment score threshold of 80 for DELTA-BLAST, and default parameters for the rest. Individual protein structure predictions were retrieved from the Alphafold website (https://alphafold.ebi.ac.uk/).

## Quantification And Statistical Analysis

Statistical analyses were performed using GraphPad Prism version 9.0.0 (Figures 1B, 1C, 1H–1M, 4B–4G, 5B, 5C, S2C, S2D, and S9A–S9C, and Protein Discovery 3.1 (Figure 2A). Details on number of replicates, error estimate, statistical tests, and significance cutoff can be found in the respective figure legends.

## Supplementary Material

Data S1

File Document S1. Figures S1–S15 and Table S4

Table S1

Table S2

Table S3

Video S1

## Data Availability

The proteomics (PXD049114), Quant-seq (PRJNA978558, PRJNA1076161), and plaNET-seq (PRJNA1076151) data is now publicly available. This paper does not report original code. Any additional information required to reanalyse the data reported in this paper is available from the lead contact upon request.

## Highlights

- APRF1, a homolog of Swd2/WDR82, is required for FCA-mediated *FLC* repression
- APRF1 in a CPF-like phosphatase module with TOPP4 (Glc7/PP1) and LD (Ref2/PNUTS)
- Loss of *APRF1* leads to transcriptional readthrough and RNA Pol II hyperphosphorylation
- APRF1 activity links co-transcriptional processing with chromatin remodeling

## References

[1] Muniz L, Nicolas E, Trouche D (2021). RNA polymerase II speed: a key player in controlling and adapting transcriptome composition. EMBO J.

[2] Žumer K, Maier KC, Farnung L, Jaeger MG, Rus P, Winter G, Cramer P (2021). Two distinct mechanisms of RNA polymerase II elongation stimulation *in vivo*. Mol Cell.

[3] Berry S, Hartley M, Olsson TSG, Dean C, Howard M (2015). Local chromatin environment of a Polycomb target gene instructs its own epigenetic inheritance. eLife.

[4] Michaels SD, Amasino RM (1999). FLOWERING LOCUS C encodes a novel MADS domain protein that acts as a repressor of flowering. Plant Cell.

[5] Macknight R, Bancroft I, Page T, Lister C, Schmidt R, Love K, Westphal L, Murphy G, Sherson S, Cobbett C, Dean C (1997). *FCA*, a gene controlling flowering time in Arabidopsis, encodes a protein containing RNA-binding domains. Cell.

[6] Xu C, Wu Z, Duan HC, Fang X, Jia G, Dean C (2021). R-loop resolution promotes co-transcriptional chromatin silencing. Nat Commun.

[7] Wu Z, Fang X, Zhu D, Dean C (2020). Autonomous pathway: *FLOWERING LOCUS C* repression through an antisense-mediated chromatin-silencing mechanism. Plant Physiol.

[8] Schon M, Baxter C, Xu C, Enugutti B, Nodine MD, Dean C (2021). Antagonistic activities of cotranscriptional regulators within an early developmental window set *FLC* expression level. Proc Natl Acad Sci USA.

[9] Liu F, Quesada V, Crevillén P, Bäurle I, Swiezewski S, Dean C (2007). The Arabidopsis RNA-binding protein FCA requires a lysine-specific demethylase 1 homolog to downregulate *FLC*. Mol Cell.

[10] Fang X, Wu Z, Raitskin O, Webb K, Voigt P, Lu T, Howard M, Dean C (2020). The 30 processing of antisense RNAs physically links to chromatin-based transcriptional control. Proc Natl Acad Sci USA.

[11] Wu Z, Ietswaart R, Liu F, Yang H, Howard M, Dean C (2016). Quantitative regulation of *FLC* via coordinated transcriptional initiation and elongation. Proc Natl Acad Sci USA.

[12] Inagaki S, Takahashi M, Takashima K, Oya S, Kakutani T (2021). Chromatin-based mechanisms to coordinate convergent overlapping transcription. Nat Plants.

[13] Sonmez C, Bäurle I, Magusin A, Dreos R, Laubinger S, Weigel D, Dean C (2011). RNA 3’ processing functions of Arabidopsis FCA and FPA limit intergenic transcription. Proc Natl Acad Sci USA.

[14] Rodríguez-Molina JB, West S, Passmore LA (2023). Knowing when to stop: transcription termination on protein-coding genes by eu-karyotic RNAPII. Mol Cell.

[15] Carminati M, Rodriguez-Molina JB, Manav MC, Bellini D, Passmore LA (2023). A direct interaction between CPF and Pol II links RNA 30-end processing to transcription. Mol Cell.

[16] Schreieck A, Easter AD, Etzold S, Wiederhold K, Lidschreiber M, Cramer P, Passmore LA (2014). RNA polymerase II termination involves C-terminal-domain tyrosine dephosphorylation by CPF subunit Glc7. Nat Struct Mol Biol.

[17] Cortazar MA, Sheridan RM, Erickson B, Fong N, Glover-Cutter K, Brannan K, Bentley DL (2019). Control of RNA Pol II speed by PNUTS-PP1 and Spt5 dephosphorylation facilitates termination by a “Sitting Duck Torpedo” mechanism. Mol Cell.

[18] Menon G, Mateo-Bonmatí E, Reeck S, Maple R, Wu Z, Ietswaart R, Dean C, Howard M (2024). Proximal termination generates a transcriptional state that determines the rate of establishment of Polycomb silencing. Mol Cell.

[19] Sanda SL, Amasino RM (1996). Ecotype-specific expression of a flowering mutant phenotype in *Arabidopsis thaliana*. Plant Physiol.

[20] Lee I, Aukerman MJ, Gore SL, Lohman KN, Michaels SD, Weaver LM, John MC, Feldmann KA, Amasino RM (1994). Isolation of *LUMINIDEPENDENS*: a gene involved in the control of flowering time in Arabidopsis. Plant Cell.

[21] Xu L, Zhao Z, Dong A, Soubigou-Taconnat L, Renou JP, Steinmetz A, Shen WH (2008). Di- and tri- but not monomethylation on histone H3 lysine 36 marks active transcription of genes involved in flowering time regulation and other processes in *Arabidopsis thaliana*. Mol Cell Biol.

[22] Qi PL, Zhou HR, Zhao QQ, Feng C, Ning YQ, Su YN, Cai XW, Yuan DY, Zhang ZC, Su XM (2022). Characterization of an autonomous pathway complex that promotes flowering in *Arabidopsis*. Nucleic Acids Res.

[23] Kapolas G, Beris D, Katsareli E, Livanos P, Zografidis A, Roussis A, Milioni D, Haralampidis K (2016). APRF1 promotes flowering under long days in Arabidopsis thaliana. Plant Sci.

[24] Fiorucci AS, Bourbousse C, Concia L, Rougée M, Deton-Cabanillas AF, Zabulon G, Layat E, Latrasse D, Kim SK, Chaumont N (2019). Arabidopsis S2Lb links AtCOMPASS-like and SDG2 activity in H3K4me3 independently from histone H2B monoubiquitination. Genome Biol.

[25] Bae HJ, Dubarry M, Jeon J, Soares LM, Dargemont C, Kim J, Geli V, Buratowski S (2020). The Set1 N-terminal domain and Swd2 interact with RNA polymerase II CTD to recruit COMPASS. Nat Commun.

[26] Casañal A, Kumar A, Hill CH, Easter AD, Emsley P, Degliesposti G, Gordiyenko Y, Santhanam B, Wolf J, Wiederhold K (2017). Architecture of eukaryotic mRNA 3’-end processing machinery. Science.

[27] Lidschreiber M, Easter AD, Battaglia S, Rodríguez-Molina JB, Casañal A, Carminati M, Baejen C, Grzechnik P, Maier KC, Cramer P, Passmore LA (2018). The APT complex is involved in non-coding RNA transcription and is distinct from CPF. Nucleic Acids Res.

[28] Jiang D, Gu X, He Y (2009). Establishment of the winter-annual growth habit via *FRIGIDA*-mediated histone methylation at *FLOWERING LOCUS C* in *Arabidopsis*. Plant Cell.

[29] Jiang D, Kong NC, Gu X, Li Z, He Y (2011). Arabidopsis COMPASS-like complexes mediate histone H3 lysine-4 trimethylation to control floral transition and plant development. PLoS Genet.

[30] Liu Y, Huang Y (2018). Uncovering the mechanistic basis for specific recognition of monomethylated H3K4 by the CW domain of *Arabidopsis* histone methyltransferase SDG8. J Biol Chem.

[31] Yang H, Howard M, Dean C (2014). Antagonistic roles for H3K36me3 and H3K27me3 in the cold-induced epigenetic switch at Arabidopsis *FLC*. Curr Biol.

[32] Simpson GG, Dijkwel PP, Quesada V, Henderson I, Dean C (2003). FY is an RNA 30 end-processing factor that interacts with FCA to control the *Arabidopsis* floral transition. Cell.

[33] Johanson U, West J, Lister C, Michaels S, Amasino R, Dean C (2000). Molecular analysis of *FRIGIDA*, a major determinant of natural variation in *Arabidopsis* flowering time. Science.

[34] Gregersen LH, Mitter R, Ugalde AP, Nojima T, Proudfoot NJ, Agami R, Stewart A, Svejstrup JQ (2019). SCAF4 and SCAF8, mRNA anti-terminator proteins. Cell.

[35] Liu F, Marquardt S, Lister C, Swiezewski S, Dean C (2010). Targeted 3^0^ processing of antisense transcripts triggers *Arabidopsis FLC* chromatin silencing. Science.

[36] Oates ME, Romero P, Ishida T, Ghalwash M, Mizianty MJ, Xue B, Dosztányi Z, Uversky VN, Obradovic Z, Kurgan L (2013). D^2^P^2^: database of disordered protein predictions. Nucleic Acids Res.

[37] Lorkovic ZJ, Park C, Goiser M, Jiang D, Kurzbauer MT, gelhofer P, Berger F (2017). Compartmentalization of DNA damage response between heterochromatin and ruchromatin is mediated by distinct H2A histone variants. Curr Biol.

[38] Jamge B, Lorković B, Axelsson E, Osakabe A, Shukla V, Yelagandula R, Akimcheva S, Kuehn AL, Berger F (2023). Histone variants shape chromatin states in Arabidopsis. eLife.

[39] Nedea E, Nalbant D, Xia D, Theoharis NT, Suter B, Richardson CJ, Tatchell K, Kislinger T, Greenblatt JF, Nagy PL (2008). The Glc7 phosphatase subunit of the cleavage and polyadenylation factor is essential for transcription termination on snoRNA genes. Mol Cell.

[40] Russnak R, Nehrke KW, Platt T (1995). REF2 encodes an RNA-binding protein directly involved in yeast mRNA 3’-end formation. Mol Cell Biol.

[41] Allen PB, Kwon YG, Nairn AC, Greengard P (1998). Isolation and characterization of PNUTS, a putative protein phosphatase 1 nuclear targeting subunit. J Biol Chem.

[42] Wang Q, Qin Q, Su M, Li N, Zhang J, Liu Y, Yan L, Hou S (2022). Type one protein phosphatase regulates fixed-carbon starvation-induced autophagy in Arabidopsis. Plant Cell.

[43] Shen L, Zhang Y, Sawettalake N (2022). A Molecular switch for *FLOWERING LOCUS C* activation determines flowering time in Arabidopsis. Plant Cell.

[44] Jumper J, Evans R, Pritzel A, Green T, Figurnov M, Ronneberger O, Tunyasuvunakool K, Bates R, Zídek A, Potapenko A (2021). Highly accurate protein structure prediction with AlphaFold. Nature.

[45] Mori S, Oya S, Takahashi M, Takashima K, Inagaki S, Kakutani T (2023). Cotranscriptional demethylation induces global loss of H3K4me2 from active genes in Arabidopsis. EMBO J.

[46] Zhou S, Zhao F, Zhu D, Zhang Q, Dai Z, Wu Z (2023). Coupling of co-transcriptional splicing and 3^0^ end Pol II pausing during termination in Arabidopsis. Genome Biol.

[47] Mikulski P, Wolff P, Lu T, Nielsen M, Echevarria EF, Zhu D, Qüesta JI, Saalbach G, Martins C, Dean C (2022). VAL1 acts as an assembly platform co-ordinating co-transcriptional repression and chromatin regulation at Arabidopsis *FLC*. Nat Commun.

[48] Xu C, Fang X, Lu T, Dean C (2021). Antagonistic cotranscriptional regulation through ARGONAUTE1 and the THO/TREX complex orchestrates *FLC* transcriptional output. Proc Natl Acad Sci USA.

[49] Castaings L, Bergonzi S, Albani MC, Kemi U, Savolainen O, Coupland G (2014). Evolutionary conservation of cold-induced antisense RNAs of *FLOWERING LOCUS C* in *Arabidopsis thaliana* perennial relatives. Nat Commun.

[50] Mo W, Liu B, Zhang H, Jin X, Lu D, Yu Y, Liu Y, Jia J, Long Y, Deng X (2021). Landscape of transcription termination in Arabidopsis revealed by single-molecule nascent RNA sequencing. Genome Biol.

[51] Kindgren P, Ivanov M, Marquardt S (2020). Native elongation transcript sequencing reveals temperature dependent dynamics of nascent RNAPII transcription in Arabidopsis. Nucleic Acids Res.

[52] Sun Q, Csorba T, Skourti-Stathaki K, Proudfoot NJ, Dean C (2013). R-loop stabilization represses antisense transcription at the Arabidopsis *FLC* locus. Science.

[53] Baxter CL, Ŝvikovic S, Sale JE, Dean C, Costa S (2021). The intersection of DNA replication with antisense 3^0^ RNA processing in *Arabidopsis FLC* chromatin silencing. Proc Natl Acad Sci USA.

[54] Landsverk HB, Sandquist LE, Bay LTE, Steurer B, Campsteijn C, Landsverk OJB, Marteijn JA, Petermann E, Trinkle-Mulcahy L, Syljuåsen RG (2020). WDR82/PNUTS-PP1 prevents transcription-replication conflicts by promoting RNA polymerase II degradation on chromatin. Cell Rep.

[55] Kirstein N, Gomes dos Santos H, Blumenthal E, Shiekhattar R (2021). The Integrator complex at the crossroad of coding and noncoding RNA. Curr Opin Cell Biol.

[56] Wagner EJ, Tong L, Adelman K (2023). Integrator is a global pro-moter-proximal termination complex. Mol Cell.

[57] Hu S, Peng L, Song A, Ji YX, Cheng J, Wang M, Chen FX (2023). INTAC endonuclease and phosphatase modules differentially regulate transcription by RNA polymerase II. Mol Cell.

[58] Liu Y, Li S, Chen Y, Kimberlin AN, Cahoon EB, Yu B (2016). snRNA 3^0^ end processing by a CPSF73-containing complex essential for development in *Arabidopsis*. PLoS Biol.

[59] Estell C, Davidson L, Eaton JD, Kimura H, Gold VAM, West S (2023). A restrictor complex of ZC3H4, WDR82, and ARS2 integrates with PNUTS to control unproductive transcription. Mol Cell.

[60] Rouvière JO, Salerno-Kochan A, Lykke-Andersen S, Garland W, Dou Y, Rathore O, Molska ES, Wu G, Schmid M, Bugai A (2023). ARS2 instructs early transcription termination-coupled RNA decay by recruiting ZC3H4 to nascent transcripts. Mol Cell.

[61] Estell C, Davidson L, Steketee PC, Monier A, West S (2021). ZC3H4 restricts non-coding transcription in human cells. eLife.

[62] Lee JH, You J, Dobrota E, Skalnik DG (2010). Identification and characterization of a novel human PP1 phosphatase complex. J Biol Chem.

[63] Russo M, Piccolo V, Polizzese D, Prosperini E, Borriero C, Polletti S, Bedin F, Marenda M, Michieletto D, Mandana GM (2023). Restrictor synergizes with Symplekin and PNUTS to terminate extragenic transcription. Genes Dev.

[64] Herr AJ, r A, Jones A, Baulcombe DC (2006). Defective RNA processing enhances RNA silencing and influences flowering of *Arabidopsis*. Proc Natl Acad Sci USA.

[65] Tian Y, Zheng H, Zhang F, Wang S, Ji X, Xu C, He Y, Ding Y (2019). PRC2 recruitment and H3K27me3 deposition at *FLC* require FCA binding of *COOLAIR*. Sci Adv.

[66] Shi Y, Di Giammartino DC, Taylor D, Sarkeshik A, Rice WJ, Yates JR, Frank J, Manley JL (2009). Molecular architecture of the human pre-mRNA 3^0^ processing complex. Mol Cell.

[67] Boreikaitė V, Passmore LA (2023). 3’-End Processing of Eukaryotic mRNA: Machinery, Regulation, and Impact on Gene Expression. Annu Rev Biochem.

[68] Li F, Cheng C, Cui F, de Oliveira MVV, Yu X, Meng X, Intorne AC, Babilonia K, Li M, Li B (2014). Modulation of RNA polymerase II phosphorylation downstream of pathogen perception orchestrates plant immunity. Cell Host Microbe.

[69] Birchler JA, Yang H (2022). The multiple fates of gene duplications: Deletion, hypofunctionalization, subfunctionalization, neofunctionalization, dosage balance constraints, and neutral variation. Plant Cell.

[70] Soares LM, Buratowski S (2012). Yeast Swd2 is essential because of antagonism between Set1 histone methyltransferase complex and APT (associated with Pta1) termination factor. J Biol Chem.

[71] Vo TV, Dhakshnamoorthy J, Larkin M, Zofall M, Thillainadesan G, Balachandran V, Holla S, Wheeler D, Grewal SIS (2019). CPF Recruitment to non-canonical transcription termination sites triggers heterochromatin assembly and gene silencing. Cell Rep.

[72] Kowalik KM, Shimada Y, Flury V, Stadler MB, Batki J, Bühler M (2015). The Paf1 complex represses small-RNA-mediated epigenetic gene silencing. Nature.

[73] Grewal SIS (2023). The molecular basis of heterochromatin assembly and epigenetic inheritance. Mol Cell.

[74] Kim M, Swenson J, McLoughlin F, Vierling E (2023). Mutation of the polyadenylation complex subunit CstF77 reveals that mRNA 3^0^ end formation and HSP101 levels are critical for a robust heat stress response. Plant Cell.

[75] Blair LP, Liu Z, Labitigan RLD, Wu L, Zheng D, Xia Z, Pearson EL, Nazeer FI, Cao J, Lang SM (2016). KDM5 lysine demethylases are involved in maintenance of 3’UTR length. Sci Adv.

[76] Kim HJ, Li P, Kim T, Oldfield AJ, Zheng X, Yang P (2022). Integrative analysis reveals histone demethylase LSD1 promotes RNA polymerase II pausing. iScience.

[77] Pinter S, Knodel F, Choudalakis M, Schnee P, Kroll C, Fuchs M, Broehm A, Weirich S, Roth M, Eisler SA (2021). A functional LSD1 coregulator screen reveals a novel transcriptional regulatory cascade connecting R-loop homeostasis with epigenetic regulation. Nucleic Acids Res.

[78] Pien S, Fleury D, Mylne JS, Crevillén P, Avramova Z, Dean C, Grossniklaus U (2008). ARABIDOPSIS TRITHORAX1 dynamically regulates *FLOWERING LOCUS C* activation via histone 3 lysine 4 trimethy-lation. Plant Cell.

[79] Weßling R, Epple P, Altmann S, He Y, Yang L, Henz SR, McDonald N, Wiley K, Bader KC, Gläßer C (2014). Convergent targeting of a common host protein-network by pathogen effectors from three kingdoms of life. Cell Host Microbe.

[80] Martin M (2011). Cutadapt removes adapter sequences from high-throughput sequencing reads. EMBnetjournal.

[81] Dobin A, Davis CA, Schlesinger F, Drenkow J, Zaleski C, Jha S, Batut P, Chaisson M, Gingeras TR (2013). STAR: ultrafast universal RNA-seq aligner. Bioinformatics.

[82] Smith T, Heger A, Sudbery I (2017). UMI-tools: modeling sequencing errors in Unique Molecular Identifiers to improve quantification accuracy. Genome Res.

[83] Bolger AM, Lohse M, Usadel B (2014). Trimmomatic: a flexible trimmer for Illumina sequence data. Bioinformatics.

[84] Tsutsui H, Higashiyama T (2017). pKAMA-ITACHI Vectors for Highly Efficient CRISPR/Cas9-Mediated Gene Knockout in *Arabidopsis thaliana*. Plant Cell Physiol.

[85] Zhu P, Lister C, Dean C (2021). Cold-induced *Arabidopsis* FRIGIDA nuclear condensates for *FLC* repression. Nature.

[86] Box MS, Coustham V, Dean C, Mylne JS (2011). Protocol: A simple phenol-based method for 96-well extraction of high quality RNA from Arabidopsis. Plant Methods.

[87] Pankow S, Bamberger C, Calzolari D, Bamberger A, Yates JR (2016). Deep interactome profiling of membrane proteins by co-inter-acting protein identification technology. Nat Protoc.

[88] Perez-Riverol Y, Bai J, Bandla C, García-Seisdedos D, Hewapathirana S, Kamatchinathan S, Kundu DJ, Prakash A, Frericks-Zipper A, Eisenacher M (2022). The PRIDE database re-sources in 2022: a hub for mass spectrometry-based proteomics evidences. Nucleic Acids Res.

[89] Kumar S, Stecher G, Li M, Knyaz C, Tamura K (2018). MEGA X: molecular evolutionary genetics analysis across computing platforms. Mol Biol Evol.

[90] Edgar RC (2004). MUSCLE: multiple sequence alignment with high accuracy and high throughput. Nucleic Acids Res.

[91] Stothard P (2000). The Sequence Manipulation Suite: JavaScript programs for analyzing and formatting protein and DNA sequences. BioTechniques.

[92] Altschul SF, Madden TL, Schäffer AA, Zhang J, Zhang Z, Miller W, Lipman DJ (1997). Gapped BLAST and PSI-BLAST: a new generation of protein database search programs. Nucleic Acids Res.

[93] Boratyn GM, Schäffer AA, Agarwala R, Altschul SF, Lipman DJ, Madden TL (2012). Domain enhanced lookup time accelerated BLAST. Biol Direct.

