# Supplementary material for: A CPF-like phosphatase module links transcription termination to chromatin silencing
Source: Mol Cell. Author manuscript; Available in PMC 2024 Jul 23. (PMC7616277; doi:10.1016/j.molcel.2024.05.016)
Supplement: File Document S1. Figures S1–S15 and Table S4 [file EMS197565-supplement-File_Document_S1__Figures_S1_S15_and_Table_S4.pdf]

Molecular Cell, Volume *84*

Supplemental information

A CPF-like phosphatase module links transcription termination to chromatin silencing

Eduardo Mateo-Bonmatí, Miguel Montez, Robert Maple, Marc Fiedler, Xiaofeng Fang, Gerhard Saalbach, Lori A. Passmore, and Caroline Dean

A CPF-like phosphatase module links transcription termination to chromatin silencing

Eduardo Mateo-Bonmatí, Miguel Montez, Robert Maple, Marc Fiedler, Xiaofeng Fang, Gerhard Saalbach, Lori A Passmore, and Caroline Dean

Supplemental Information

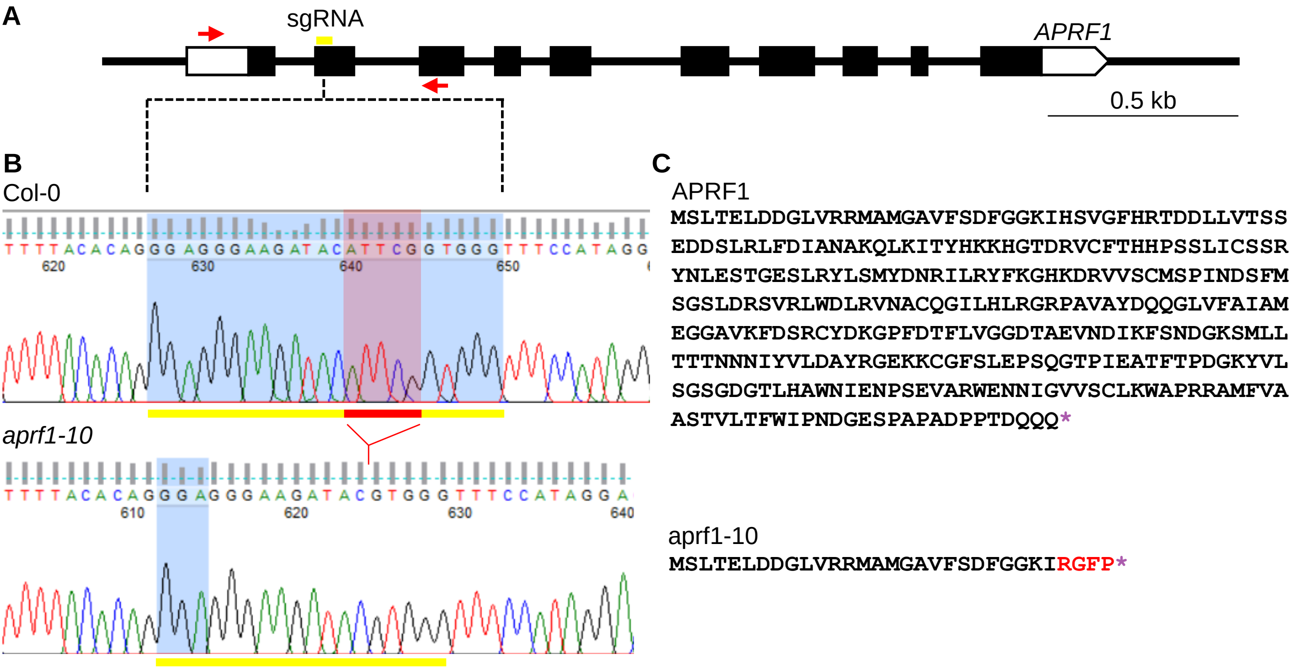

Figure S1. Details of the *APRF1* allele obtained by CRISPR-Cas9. (A) Structure of the *APRF1* gene with indication of the position of the single guide RNA (sgRNA, horizontal yellow bar, not drawn to scale) used to edit *APRF1*. Horizontal red arrows represent oligonucleotides (not drawn to scale) used to amplify and sequence the region. (B) Electropherogram highlighting in blue the sgRNA sequence in Col-0 and in red the 5-nt deletion found in the *aprf1-10* plants. (C) Predicted effects on the APRF1 protein caused by the *aprf1-10* mutation. The deletion change the protein frame keeping intact only the first 27 (of 328) amino acids (aa), then introducing 4 aa not present in the wild-type and finally creating a premature stop codon, truncating the protein with only 31 aa. Related to Figure 1.

Mateo-Bonmatí et al. Figure S2

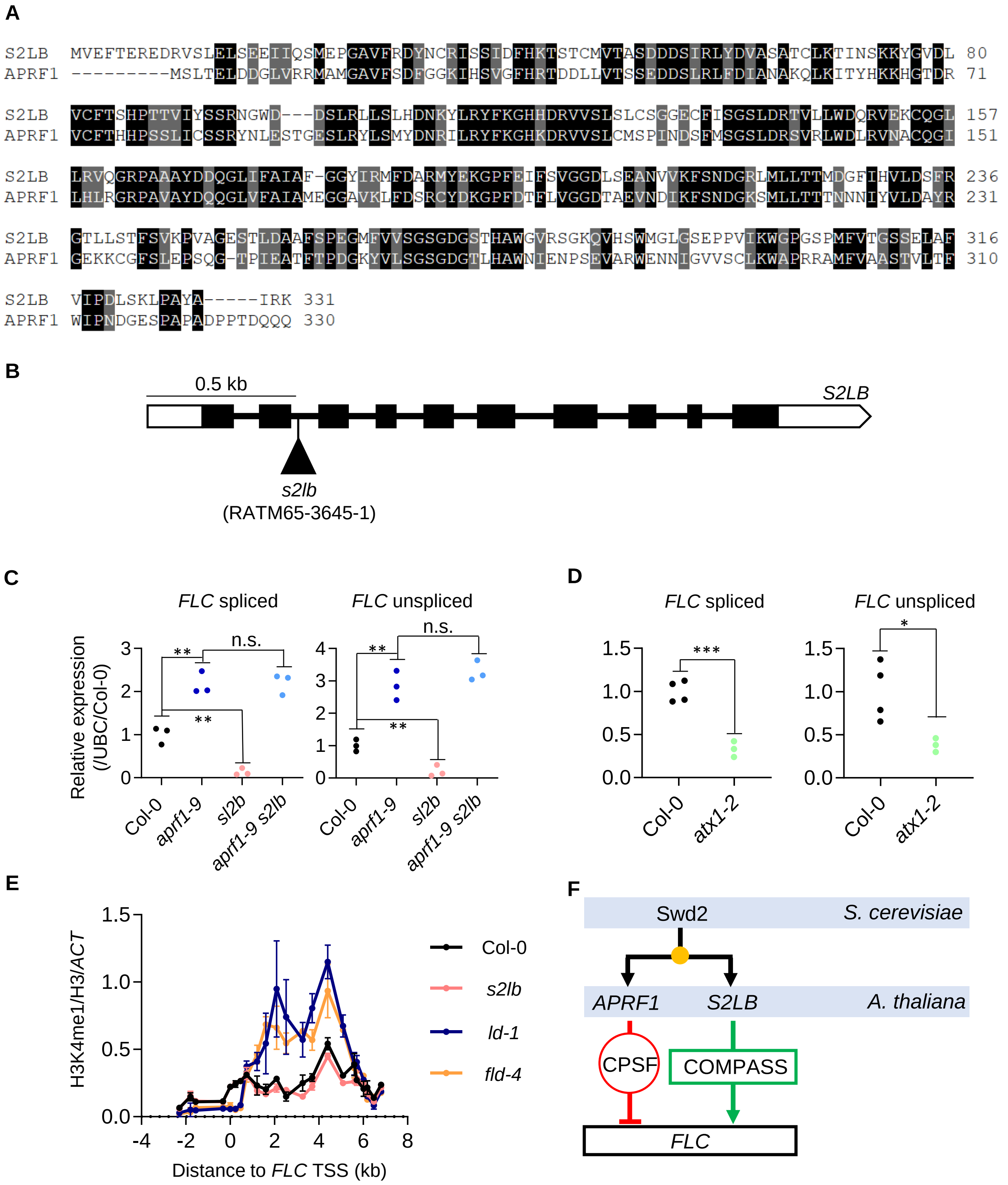

Figure S2. The Arabidopsis Swd2 orthologs seem to be sub-functionalized.
(A) Alignment of the amino acid sequences of the two Arabidopsis Swd2 orthologs: APRF1 and S2LB. Identical and similar residues are shaded in black and grey, respectively. Numbers indicate amino acid positions. Protein sequences retrieved from NCBI [APRF1 (NP_196957.1), S2LB (NP_569031.1)], aligned with MUSCLE, and shaded with the Colour Align Conservation tool from the Sequences Manipulation Suite (https://www.bioinformatics.org/sms2/color_align_cons.html).
(B) Structure of the *S2LB* gene with indication of the position of the T-DNA mutation analysed.
(C, D) Relative values of *FLC* spliced (left) and unspliced (right) in the (C) single mutants *aprf1-9, s2lb,* and the double mutant *aprf1-9 s2lb,* and (D) and *atx1-2* compared to the wild-type Col-0. Values were normalized to the housekeeping *UBC* gene and to Col-0. Asterisks indicate statistically significant differences to Col-0 in a Student's t test (* p < 0,05; ** p < 0,01; *** p < 0,001; n≥3). Each replicate consists in a pool of 10-to-15 2-week old seedlings grown in long day conditions. N.s. stands for not statistically different.
(E) ChIP analysis of H3K4me1 levels at *FLC* in Col-0, *s2lb*, *fld-4* and *ld-1*. Numbers in x axis represent the distance in kilobases to the *FLC* TSS and numbers in the y axis correspond to relative enrichment of the corresponding histone mark. Each dot represents an amplicon, analysed in three biological replicates. Values were normalized to H3 and to *ACT* and represent mean ± standard error of the mean (s.e.m.). Each replicate consists in 2.5 gr of 2-week-old seedlings.
(F) Subfunctionalization model by which the ancestral Swd2 in yeast, found to work both in the CPSF (CPF in yeast) and the COMPASS complexes, gave rise to two Arabidopsis paralogs (*APRF1* and *S2LB*), opposingly influencing the *FLC* transcriptional output by working through independent pathways. The yellow dot represents the subfunctionalization event. Related Figure 1.

A

BLASTP against Arabidopsis proteome using *H. sapiens* PNUTS as Query

Accession	AGI Code	Protein Name	Max Score	Total Score	Query Cover (%)	E value	% Of identity
NP_001190660.1	At4g02560	LUMINIDEPENDENS	48.9	48.9	7	2e-05	27.4

B

DELTA-BLAST against Arabidopsis proteome using *H. sapiens* PNUTS as Query (alignment score threshold 80)

Accession	AGI Code	Protein Name	Max Score	Total Score	Query Cover (%)	E value	% Of identity
NP_001190660.1	At4g02560	LUMINIDEPENDENS	81.6	81.6	41	3e-15	21.16

C

PSI-BLAST against Arabidopsis proteome using *H. sapiens* PNUTS as Query

Accession	AGI Code	Protein Name	Max Score	Total Score	Query Cover (%)	E value	% Of identity
NP_001190660.1	At4g02560	LUMINIDEPENDENS	48.9	48.9	7	2e-05	27.4

Figure S3. Different Protein BLAST algorithms indicate that LD is the Arabidopsis ortholog of PNUTS. Sequence of the human PNUTS (NP_001363124) was used as a query to find similar proteins in the Arabidopsis proteome by using a (A) BLASTP, (B) DELTA-BLAST, and (C) PSI-BLAST. BLASP retrieved only one matching protein. DELTA- and PSI-BLAST found only LD applying a Max Score threshold of 80 (for DELTA-BLAST) and the default threshold (for PSI-BLAST). Related to Figure 2.

Mateo-Bonmatí et al. Figure S4

A

```
PNUTS ---------------------------------------------MGSGPID---------------PKELLK------- 13
LD    MDAFKEEIEIGSSVESLMELLDSQKVLFHSQIDQLQDVVVAQCKLTGVNPLAQEMAAGALSIKIGKRPRDLLNPKAVKYL 80
Ref2  ----------------------------------------------MSAPVP-----------------QLVN------- 10

PNUTS ----------------------GL------DSFLNRDGEVKSV------------------DGISK-------------- 33
LD    QAVFAIKDAISKRESREISALFGITVAQVREFFVTQKTRVRKQVRLSREKVVMSNTHALQDDGVPENNNATNHVEPVPLN 160
Ref2  ------------------------------ISHALQASTIQQI------------------------------------- 23

PNUTS ---------------------------------------IFSLMKEARKMVSRCTYLNILLQTRSPEILVKFIDVGGYKL 74
LD    SIHPEACSISWGEGETVALIPPEDIPPDISDSDKYFVENIFSLLRKEETFSGQVKLMEWIMQIQDASVLIWFLSKGGVLI 240
Ref2  ---------------------------------------RLDMV----DFNKDCKLSSIQL------------------- 41

PNUTS LNNWLTYSKTTNNIPLLQQILLTLQHLPLTVDHLKQNNTAKLVKQLS--KSSEDEELRKLASVLVSDWMAVIRSQSSTQP 152
LD    LTTWLSQAASEEQTSVLLLILKVLCHLPL--HKASPENMSAILQSVNGLRFYRISDISNRAKGLLSRWTKLF---AKIQA 315
Ref2  -------ARIDKYIDSLQAALNQFTKDNLHIERKEKNVTEADIQLYSGLKSMYLDYLNQLIK------------------ 96

PNUTS AEKDKKKRKDEGKSRTTLPERPLTEVKAE-TRAEEAPEKKREKPKSLRTTAPSHAKFRSTGLELETPSLVPVKKNASTVV 231
LD    MKKQNRNSSQIDSQSQLLLKQSIAEIMGDSSNPEDILSLSNGKSENVRRIESSQG-----------PKLLLTSADDST-- 382
Ref2  LKHEKQHHSTPPIANDVSLDFFVNQL------PKFSPEERKNYIDNLILNKNSHNR--------------LSKMDGLV-- 154

PNUTS VSDKYNLKPIPL-----KRQSNVAAPGDATPPAEKKYKPINTTPNATKEIKVKIIPPQPMEGLGFLDALNSAPVPGIKIK 306
LD    --KKHMLGSNPSYNKERRKVQMVEQPGQKAAGKSPQTVRIGTS-GRSRPMSADDIQKAKMRAL-YMQSKNSKKDPLPSAI 458
Ref2  -----------------DAVINLCVLDTSVAENVRSYMKL-------------------LDTLGFQKGSNST---GTKAN 195

PNUTS KKKKVLSPTAA-KPSPFEGKTSTEPSTAK-PSSPEPAPPSEAMDADRPGTPVPPVEVPELMDTASLEPGALDAKPVESPG 384
LD    GDSKIVAP--E-KPLALHSAKDSPPIQNN-EAKTEDTPVLSTVQPVNGFSTIQPVNGPSAVQPVN---GPLAVQPVNGPS 531
Ref2  LKKKLASSKAKIKDSEKEKEKEKDKSKVKMKTKLKPSPLLN--NDDKNSSPSPTASTSSMKKLKS---GLFNKNEAKSTE 270

PNUTS DPNQLTRKGRKRKSVTWPEEGKLREYF--YFELDETERVNVNKIKDFGEAAKREILSDRHAFETARRLSHDNMEEKVPWV 462
LD    ALQ--------------PVNGPSAVIV--PVQADEIKKPSTPPKSISSKVGVMMKMSSQTILKNCKR-------KQIDWH 588
Ref2  SL---------------PTSSKKKLSFSKYLNKDDAD---------------MTKLGTKRSID-------------VDFK 307

PNUTS C-PRPLVLPSPL-VTPGSNSQERYIQAEREK-------GILQELFLNKESPHEPD--------PEPYEPIPPKLIPLDEE 525
LD    V-PPGMELDELWRVAAGGNSKEADVQRNRNRRERETTYQSLQTIPLNPKEPWDREMDYDDSLTPEIPSQQPPEESLTEPQ 667
Ref2  VNPEASTVASNIISSSTSGSSTTTVATPASS-----------EEPLKKKTKISVQ----DSNVQSILRNGKPKKARISSI 372

PNUTS CSMDETPYVETLEPGGSGGSPDGAGGSKLPPVLAN--LMGSMGAGKGPQGPGGGGINVQEILTSIMGSPNSHPSEELLKQ 603
LD    DSLDERRIAAGAATTSSSLSSPEPDLELLAALLKNPDLVYALTSGKPSNLAGQDMVKLLDVIKT--GAPNSSSS------ 739
Ref2  KFLDDSQLIKVY-----GDDLPNQGLQVSPTQLKK--ILKPFKEGEP-----------KEIILF---------------- 418

PNUTS PDYSDKIKQMLVPHGLLGPGPIANGFPPGGPG-GPKGMQHFPPGPGGPMPGPHGGPGGPVGPRLLGPPPPPRGGDPFWDG 682
LD    ---SNKQVEERVEVSLPSPTPSTN---PGMSGWGQEGIRN-------PFSRQN-----QVGTAVARSGTQLRVGSMQWHQ 801
Ref2  ---EDMSIKLK-----------------------------------------------PLDLMFLKN-----------TN 437

PNUTS PGDPMRGGPMRGGPGPG----PGPY-------HRGRGGRGGNEPPPPPPPFRGARGGRSGGGPPNGRGGPGGGMVGGGGH 751
LD    TNE-------QSIPRHA----PSAYSNSITLAHTEREQQQYMQPKLH--------------------------------H 838
Ref2  SDDYMDISETKGGPIHCETRTPLIY----------RKNFNHFNPDLN--------------------------------- 474

PNUTS RPHEGPGGGMGNSSGHRPHEGPGGGMGSGHRPHEGPGGSMGGGGGHRPHEGPGGGISGGSGHRPHEGPGGGMGAGGGHRP 831
LD    NLHFQQQQQQPISTTSYAVREPVGQMGTGTSSSWRSQQSQNSYYSHQENE-----IASASQVTSYQGNSQYMSSNPGYES 913
Ref2  ---------------KRPPREPI-------------EFDLNGNTNSTPTI-----------AKAFGKNSLLLRKDRGGLP 515

PNUTS HEGPGGSMGGSGGHRPHEGPGHGGPHGHRPHDVPGHRGHDHRGPPPHEHRGHDGPGHGGGGHRGHDGGHSHGGDMSNRPV 911
LD    WS---------------------------PDNSPSRNQLNMRGQQQQASRKHDSSTH----------------------- 943
Ref2  YK-----------------------------HVPIVKRNKYPPRPVH--------------------------------- 533

PNUTS CRHFMMKGNCRYENNCAFYHPGVNGPPLP 940
LD    ----------PYWNQNKRWR--------- 953
Ref2  ----------------------------- 533
```

B

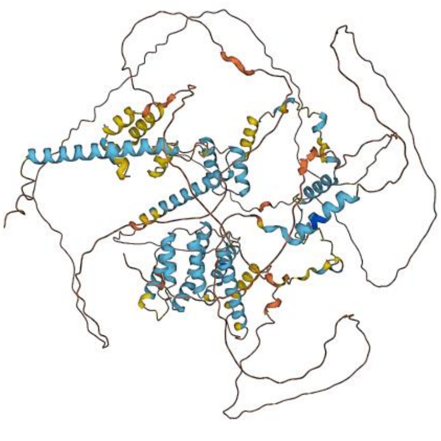

LUMINIDEPENDENS

C

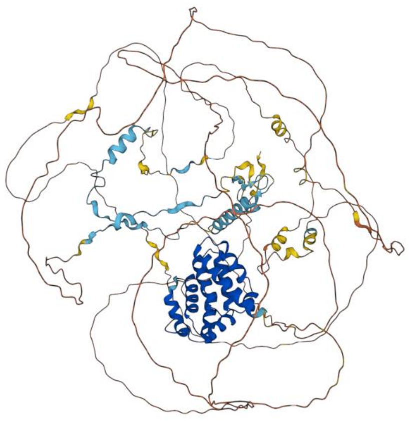

PNUTS

D

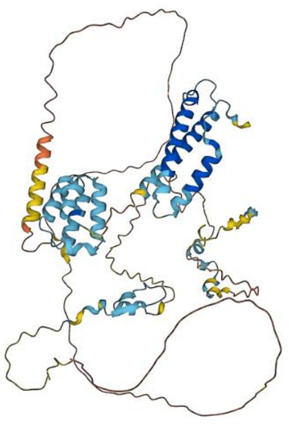

Ref2

Figure S4. Alignment of the amino acid sequences of the LD (Arabidopsis), PNUTS (Human) and Ref2 (Yeast) proteins. Identical and similar residues are shaded in black and grey, respectively. Numbers indicate amino acid positions. Protein sequences retrieved from NCBI [LD (NP_001190660.1), PNUTS (NP_001363124), Ref2 (QHB07655)], aligned with MUSCLE, and shaded with the Colour Align Conservation tool from the Sequences Manipulation Suite (https://www.bioinformatics.org/sms2/color_align_cons.html). 3D structure prediction of (B) the LD, (C) PNUTS, and (D) Ref2. The structure was downloaded from AlphaFold Protein Structure Database (https://alphafold.ebi.ac.uk/). Related to Figure 2.

```
PNUTS-HS ---------------------------------------------MGSGPID---------------PKELLK------- 13
LD       MDAFKEEIEIGSSVESLMELLDSQKVLFHSQIDQLQDVVVAQCKLTGVNPLAQEMAAGALSIKIGKRPRDLLNPKAVKYL 80

PNUTS-HS ----------------------GL------DSFLNRDGEVKSV------------------DGISK-------------- 33
LD       QAVFAIKDAISKRESREISALFGITVAQVREFFVTQKTRVRKQVRLSREKVVMSNTHALQDDGVPENNNATNHVEPVPLN 160

PNUTS-HS ---------------------------------------IFSLMKEARKMVSRCTYLNILLQTRSPEILVKFIDVGGYKL 74
LD       SIHPEACSISWGEGETVALIPPEDIPPDISDSDKYFVENIFSLLRKEETFSGQVKLMEWIMQIQDASVLIWFLSKGGVLI 240

PNUTS-HS LNNWLTYSKTTNNIPLLQQILLTLQHLPLTVDHLKQNNTAKLVKQLS--KSSEDEELRKLASVLVSDWMAVIRSQSSTQP 152
LD       LTTWLSQAASEEQTSVLLLILKVLCHLPL--HKASPENMSAILQSVNGLRFYRISDISNRAKGLLSRWTKLF---AKIQA 315

PNUTS-HS AEKDKKKRKDEGKSRTTLPERPLTEVKAE-TRAEEAPEKKREKPKSLRTTAPSHAKFRSTGLELETPSLVPVKKNASTVV 231
LD       MKKQNRNSSQIDSQSQLLLKQSIAEIMGDSSNPEDILSLSNGKSENVRRIESSQG-----------PKLLLTSADDST-- 382

PNUTS-HS VSDKYNLKPIPL-----KRQSNVAAPGDATPPAEKKYKPLNTTPNATKEIKVKIIPPQPMEGLGFLDALNSAPVPGIKIK 306
LD       --KKHMLGSNPSYNKERRKVQMVEQPGQKAAGKSPQTVRIGTS-GRSRPMSADDIQKAKMRAL-YMQSKNSKKDPLPSAI 458

PNUTS-HS KKKKVLSPTAAKPSPFEGKTSTEPSTAKPSSPEPAPPSEAMDADRPGTPVPPVEVPELMDTASLEPGALDAKPVESPGDP 386
LD       GDSKIVAP--EKPLALHSAKDSPPIQNNEAKTEDTPVLSTVQPVNGFSTIQPVNGPSAVQPVN---GPLAVQPVNGPSAL 533

PNUTS-HS NQLTRKGRKRKSVTWPEEGKLREYFYFELDETERVNVNKIKDFGEAAKREILSDRHAFETARRLSHDNMEEKVPWVCPRP 466
LD       Q--------------PVNGPSAVIVPVQADEIKKPSTPPKSISSKVGVMMKMSSQTILKNCKR-------KQIDWHVPPG 592

PNUTS-HS LVLPSPL-VTPGSNSQERYIQAEREK-------GILQELFLNKESPHEPD--------PEPYEPIPPKLIPLDEECSMDE 530
LD       MELDELWRVAAGGNSKEADVQRNRNRRERETTYQSLQTIPLNPKEPWDREMDYDDSLTPEIPSQQPPEESLTEPQDSLDE 672

PNUTS-HS TPYVETLEPGGSGGSPDGAGGSKLPPVLAN--LMGSMGAGKGPQGPGGGGINVQEILTSIMGSPNSHPSEELLKQPDYSD 608
LD       RRIAAGAATTSSSLSSPEPDLELLAALLKNPDLVYALTSGKPSNLAGQDMVKLLDVIKT--GAPNSSSS---------SN 741

PNUTS-HS KIKQMLVPHGLLGPGPIANGFPPGGPG-GPKGMQHFPPGPGGPMPGPHGGPGGPVGPRLLGPPPPPRGGDPFWDGPGDPM 687
LD       KQVEERVEVSLPSPTPSTN---PGMSGWGQEGIRN-------PFSRQN-----QVGTAVARSGTQLRVGSMQWHQTNE-- 804

PNUTS-HS RGGPMRGGPGPGPGPY-------HRGRGGRGGNEPPPPPPPFRGARGGRSGGGPPNGRGGPGGGMVGGGGHRPHEGPGGG 760
LD       -----QSIPRHAPSAYSNSITLAHTEREQQQYMQPKLH--------------------------------HNLHFQQQQQ 847

PNUTS-HS MGNSSGHRPHEGPGGGMGSGHRPHEGPGGSMGGGGGHRPHEGPGGGISGGSGHRPHEGPGGGMGAGGGHRPHEGPGGSMG 840
LD       QPISTTSYAVREPVGQMGTGTSSSWRSQQSQNSYYSHQENE-----IASASQVTSYQGNSQYMSSNPGYESWS------- 915

PNUTS-HS GSGGHRPHEGPGHGGPHGHRPHDVPGHRGHDHRGPPPHEHRGHDGPGHGGGGHRGHDGGHSHGGDMSNRPVCRHFMMKGN 920
LD       --------------------PDNSPSRNQLNMRGQQQQASRKHDSSTH-------------------------------- 943

PNUTS-HS CRYENNCAFYHPGVNGPPLP 940
LD       -PYWNQNKRWR--------- 953
```

Figure S5. Alignment of the amino acid sequences of the LD (Arabidopsis), and PNUTS (Human). Identical and similar residues are shaded in black and grey, respectively. Numbers indicate amino acid positions. Protein sequences retrieved from NCBI [LD (NP_001190660.1), and PNUTS (NP_001363124)], aligned with MUSCLE, and shaded with the Colour Align Conservation tool from the Sequences Manipulation Suite (https://www.bioinformatics.org/sms2/color_align_cons.html). Related to Figure 2.

Mateo-Bonmatí et al. Figure S6

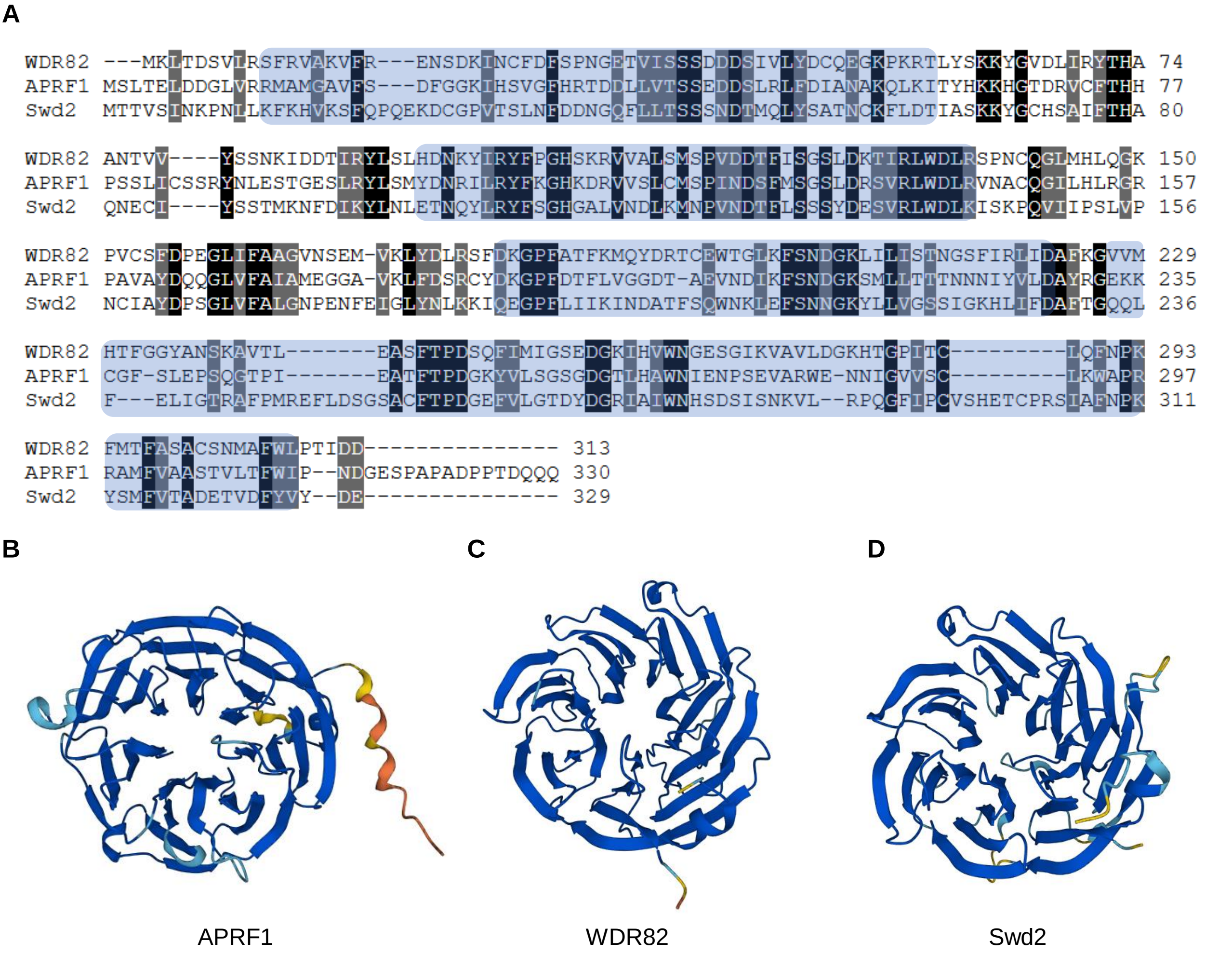

Figure S6. Alignment of the amino acid sequences of the APRF1 (Arabidopsis), WDR82 (Human) and Swd2 (Yeast) proteins. Identical and similar residues are shaded in black and grey, respectively. Numbers indicate amino acid positions. Protein sequences retrieved from NCBI [APRF1 (NP_196957.1), WDR82 (NP_079498.2), Swd2 (NP_012907.3)], aligned with MUSCLE, and shaded with the Colour Align Conservation tool from the Sequences Manipulation Suite (https://www.bioinformatics.org/sms2/color_align_cons.html). Highlighted in pale blue the conserved WD40-repeat domain (InterPro 001680). 3D structure prediction of (B) the Arabidopsis APRF1, (C) the human WDR82, and (D) the yeast Swd2. Structures were downloaded from AlphaFold Protein Structure Database (https://alphafold.ebi.ac.uk/). Related to Figure 2.

A

```
PP1    ----------MSDSEKLNLDSIIGRLLEVQGSRPGKNVQLTENEIRGLCLKSREIFLSQPILLELEAPLKICGDIHGQYY 70
TOPP4  MATTTTTQGQQTAIDSAVLDDIIRRLTEVRLARPGKQVQLSEAEIKQLCTTARDIFLQQPNLLELEAPIKICGDIHGQYS 80
Glc7   -----------MDSQPVDVDNIIDRLLEVRGSKPGQQVDLEENEIRYLCSKARSIFIKQPILLELEAPIKICGDIHGQYY 69

PP1    DLLRLFEYGGFPPESNYLFLGDYVDRGKQSLETICLLLAYKIKYPENFFLLRGNHECASINRIYGFYDECKRRYNIKLWK 150
TOPP4  DLLRLFEYGGFPPSANYLFLGDYVDRGKQSLETICLLLAYKIKYPGNFFLLRGNHECASINRIYGFYDECKRRFNVRVWK 160
Glc7   DLLRLFEYGGFPPESNYLFLGDYVDRGKQSLETICLLLAYKIKYPENFFILRGNHECASINRIYGFYDECKRRYNIKLWK 149

PP1    TFTDCFNCLPIAAIVDEKIFCCHGGLSPDLQSMEQIRRIMRPTDVPDQGLLCDLLWSDPDKDVQGWGENDRGVSFTFGAE 230
TOPP4  VFTDCFNCLPVAALIDDKILCMHGGLSPDLDHLDEIRNLPRPTMIPDTGLLCDLLWSDPGKDVKGWGMNDRGVSYTFGPD 240
Glc7   TFTDCFNCLPIAAIIDEKIFCMHGGLSPDLNSMEQIRRVMRPTDIPDVGLLCDLLWSDPDKDIVGWSENDRGVSFTFGPD 229

PP1    VVAKFLHKHDLDLICRAHQVVEDGYEFFAKRQLVTLFSAPNYCGEFDNAGAMMSVDETLMCSFQILKPADKNKGKYGQFS 310
TOPP4  KVSEFLTKHDLDLVCRAHQVVEDGYEFFADRQLVTVFSAPNYCGEFDNAGAMMSVDENLMCSFQILKPAEKKTKF----- 315
Glc7   VVNRFLQKQDMELICRAHQVVEDGYEFFSKRQLVTLFSAPNYCGEFDNAGAMMSVDESLLCSFQILKPAQKSLPR----- 304

PP1    GLNPGGRPITPPRNSAKAKK 330
TOPP4  --------------MMSTKI 321
Glc7   --QAGGR---------KKKX 313
```

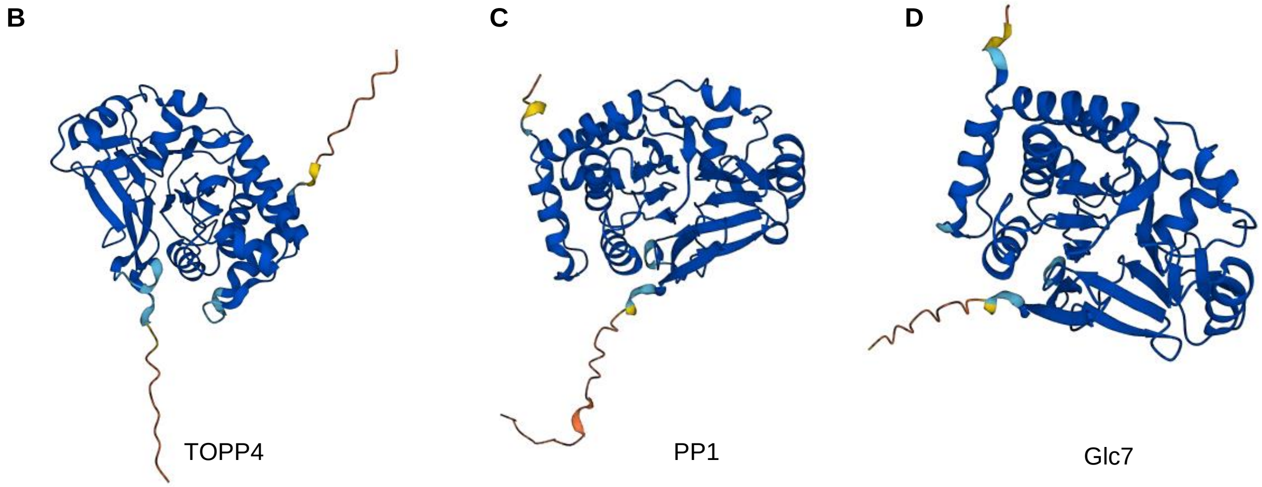

Figure S7. Alignment of the amino acid sequences of the TOPP4 (Arabidopsis), PP1 (Human) and Glc7 (Yeast) proteins. Identical and similar residues are shaded in black and grey, respectively. Numbers indicate amino acid positions. Protein sequences retrieved from NCBI [TOPP4 (OAP08593.1), PP1 (NP_002699.1), Glc7 (NP_011059.3)], aligned with MUSCLE, and shaded with the Colour Align Conservation tool from the Sequences Manipulation Suite (https://www.bioinformatics.org/sms2/color_align_cons.html). Highlighted in pale purple the conserved Serine/threonine-specific protein phosphatase/bis(5-nucleosyl)-tetraphosphatase domain (InterPro 006186). 3D structure prediction of (B) the Arabidopsis TOPP4, (C) the human PP1, and (D) the yeast Glc7. Structures were downloaded from AlphaFold Protein Structure Database (https://alphafold.ebi.ac.uk/). Related to Figure 2.

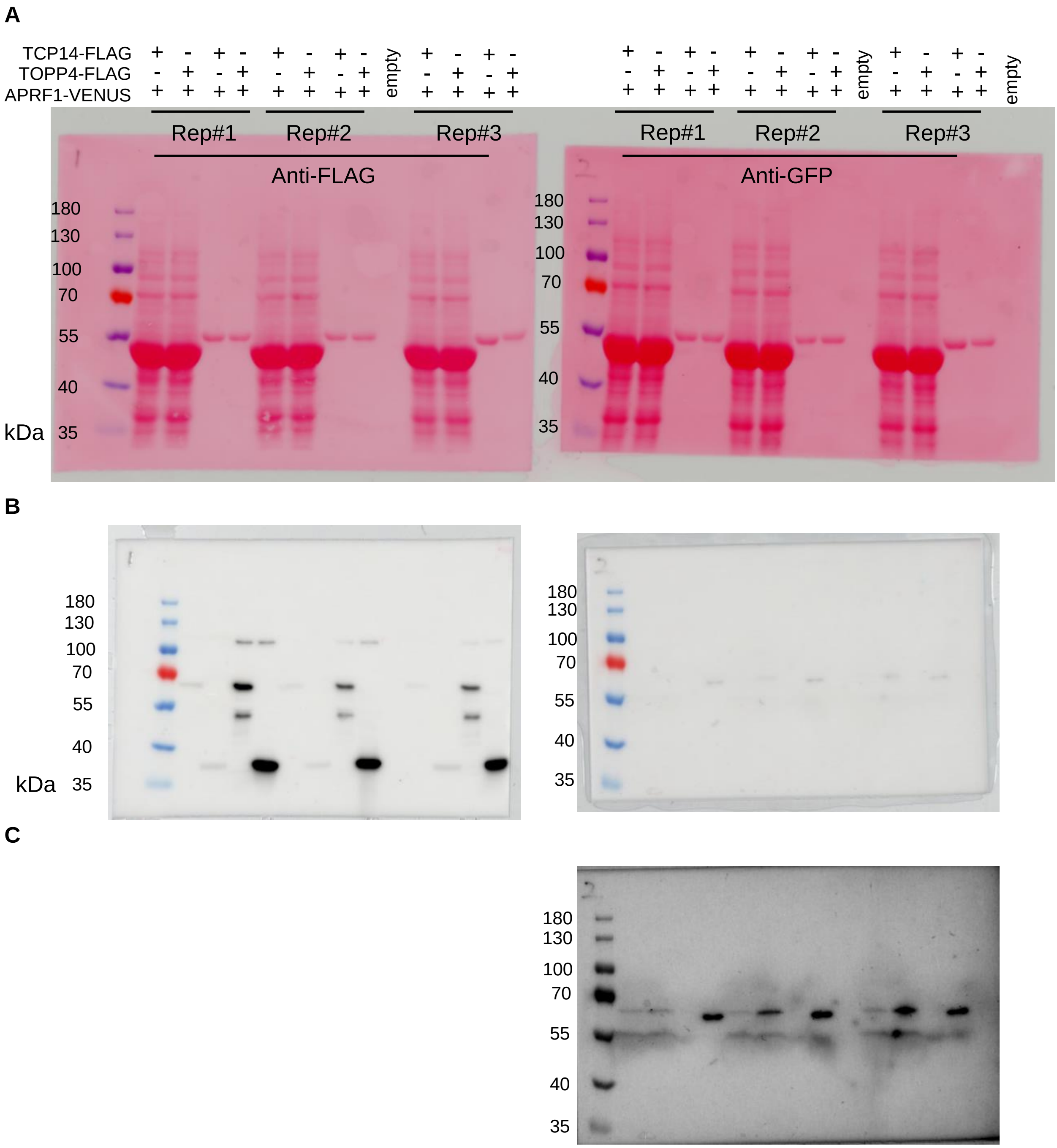

Figure S8.Uncropped blots from *N. benthamiana* co-IP experiments.
(A) Ponceau staining of anti-FLAG and anti-GFP blots before primary antibody hybridization. Sample contained in each well is defined in the legend above the blots. Protein ladder is shown in the first left well of each blot. Expected sizes of ladder bands are indicated for all blots in kDa (PageRuler™ Prestained Protein Ladder, ThermoFisher Scientific, ref: 26616). (B, C) Western blot results of the membranes shown above using as substrate SuperSignal West Pico (B), and SuperSignal West Femto (C). Expected protein sizes are 62, 52, and 39 kDa for APRF1-VENUS, TCP14-FLAG, and TOPP4-FLAG, respectively. Related to Figure 2 and STAR Methods.

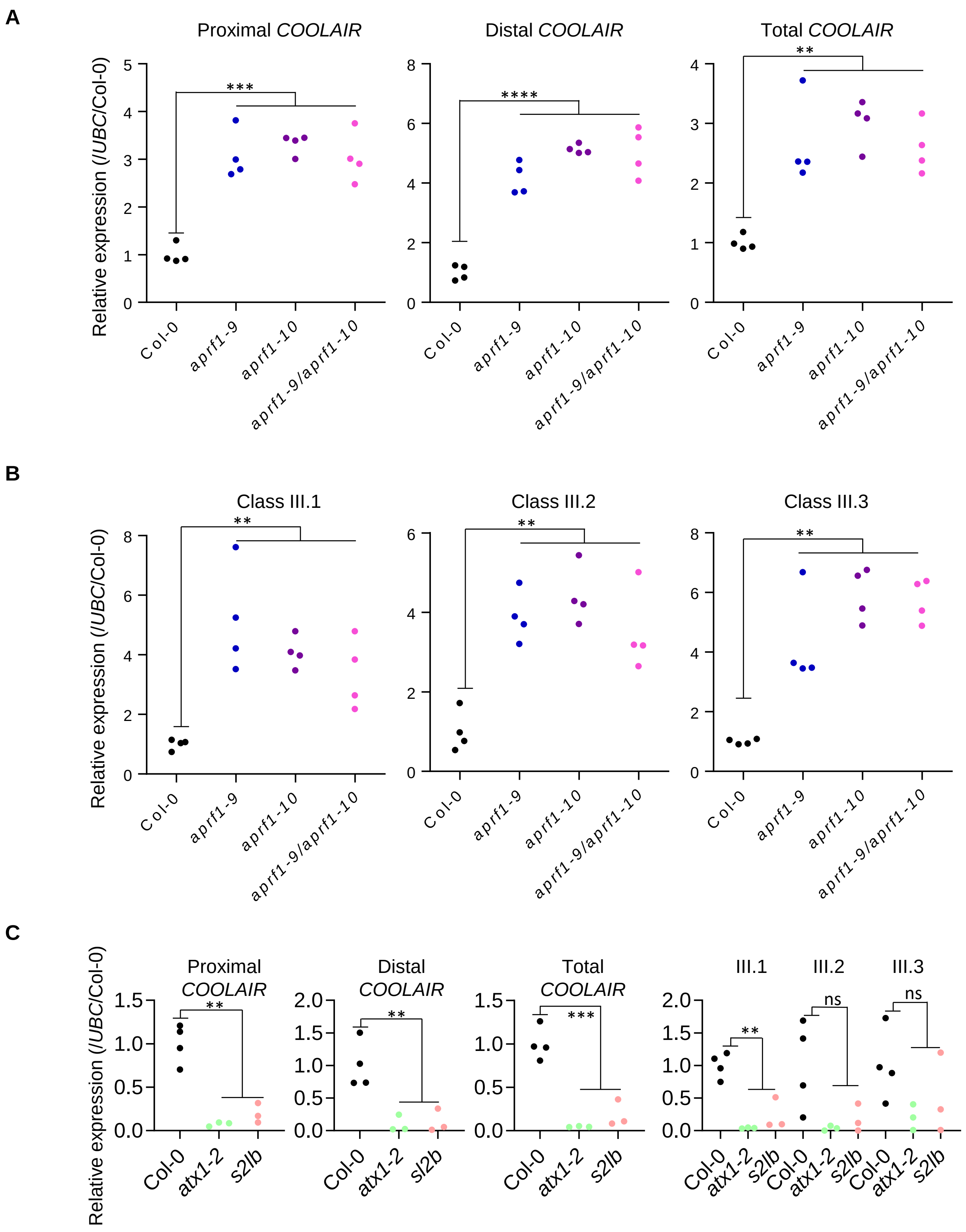

Figure S9. Full *COOLAIR* profile of the different *aprf1* alleles and the COMPASS-related mutant. Relative values of the indicated *COOLAIR* isoform in the wild-type Col-0, (A, B) the homozygous *aprf1-9/aprf1-9*, *aprf1-10/aprf1-10,* and the hybrid *aprf1-9/aprf1-10* plants, and (C) the *atx1-2* and *s2lb* homozygous mutants. Values were normalized to the housekeeping *UBC* gene and to Col-0. Asterisks indicate statistically significant differences to Col-0 in a Student's *t* test (ns, not significant; ** *p* < 0,01; *** *p* < 0,001; **** *p* < 0,0001; n ≥ 3). Experiments were performed in 2-week-old seedlings (A, B) or 10-day old seedlings (C) grown in long days conditions. Each replicate consists in a pool of 10-to-15 seedlings. Related to Figure 4.

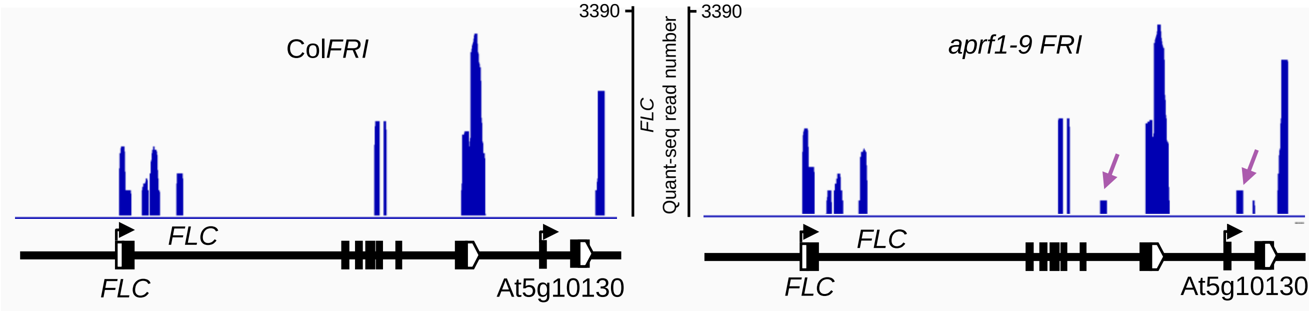

Figure S10. Quant-seq results from Col*FRI* and *aprf1-9 FRI* on the sense strand of *FLC*. Purple arrows point to reads detecting polyadenylation sites found in *aprf1-9 FRI* absent in Col*FRI*. Related to Figure 4.

Mateo-Bonmatí et al. Figure S11

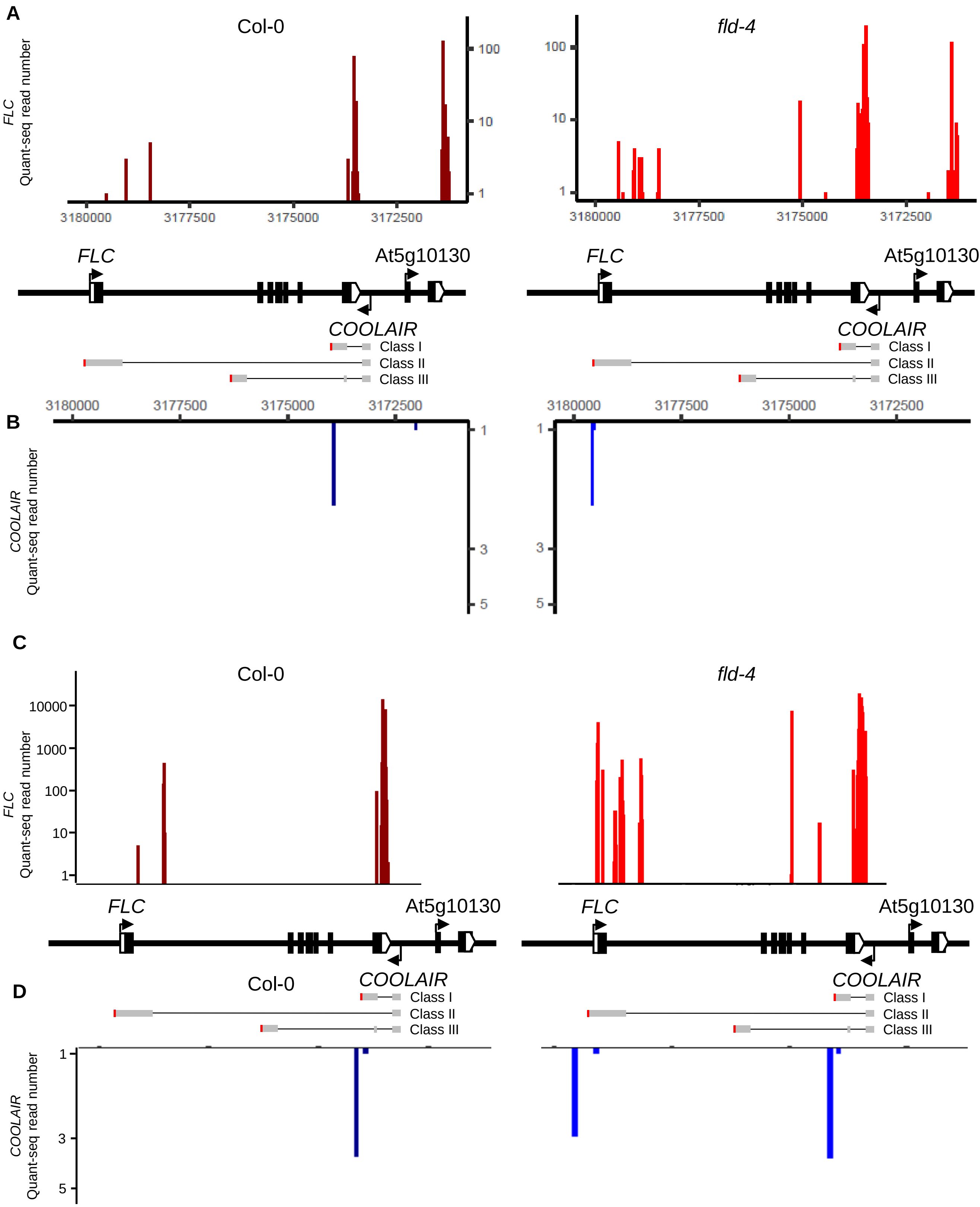

Figure S11. Quant-seq analysis on *FLC/COOLAIR* of the *fld-4* mutant. (A, B) Standard Quant-seq signal over the *FLC* and *COOLAIR* strands using total RNA to prepare the library. On the left, the wild-type Col-0, on the right, the mutant *fld-4*. (C, D). Bait-enriched Quant-seq libraries signal over the same locus following the same structure. A 100-fold increase in the *FLC* signal can be appreciated after the bait enrichment. Related to Figure 4.

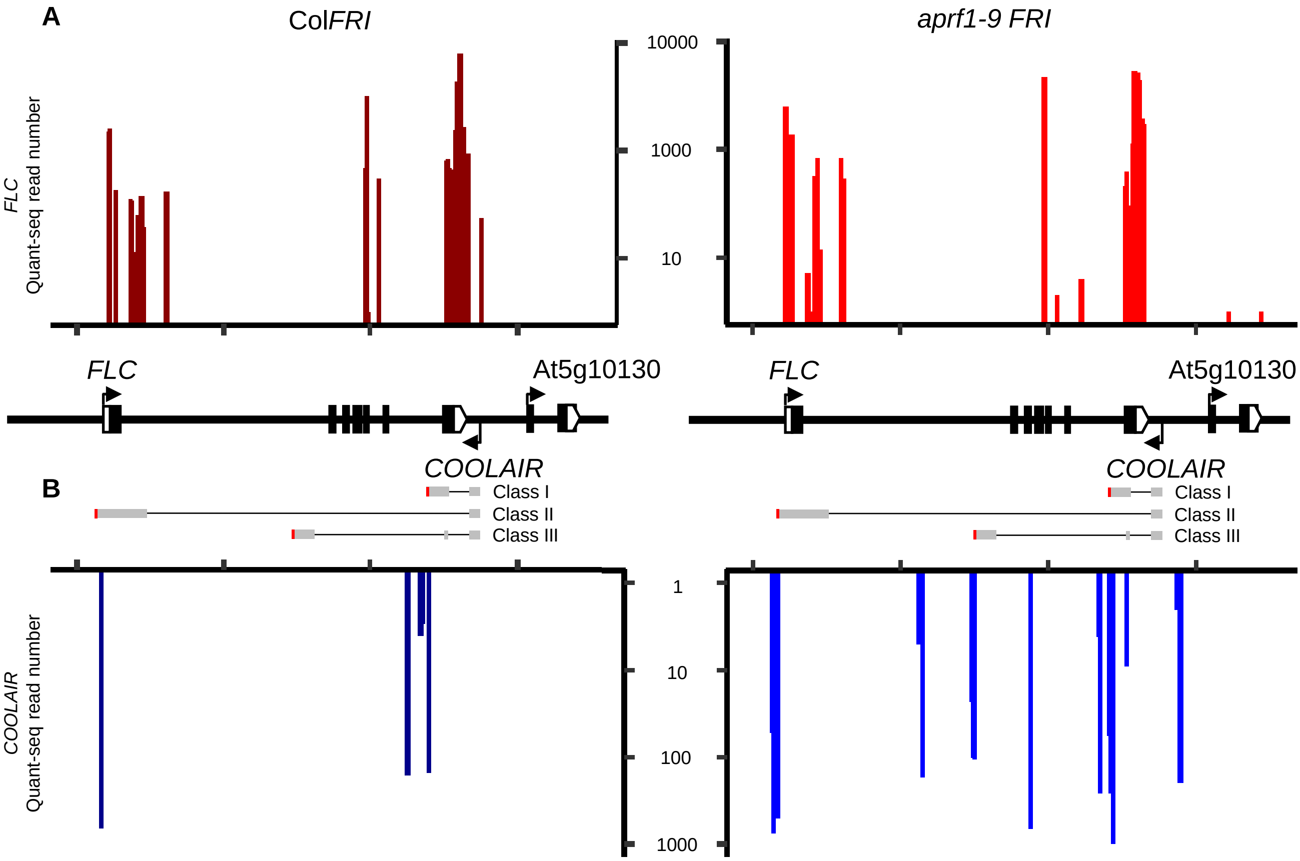

Figure S12. Quant-seq analysis after bait-enrichment in the *aprf1-9 FRI* mutant. (A, B) Bait-enriched Quant-seq signal over the *FLC* locus (A) sense, (B) antisense, in Col*FRI* (left), and *aprf1-9 FRI* (right). Related to Figure 4.

Mateo-Bonmatí et al. Figure S13

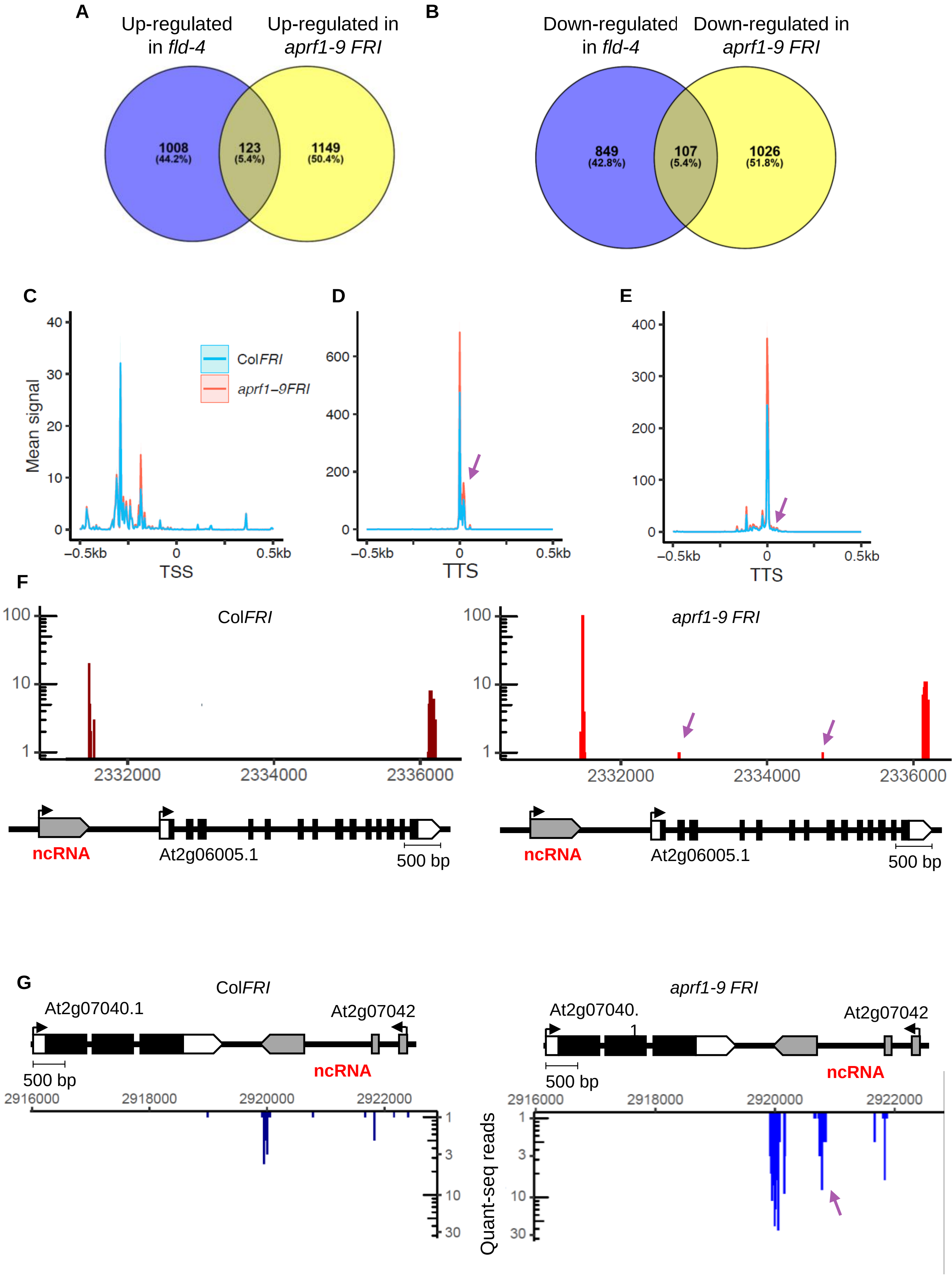

Figure S13. Analysis of the genes co-regulated by APRF1 and FLD. (A, B) Venn diagrams showing the overlapping between upregulated (A), and downregulated (B) gene sets in *fld-4* and *aprf1 FRI* compared to the respective wild-type strain. (C, D) Quant-seq signal metaplot of Col*FRI* and *aprf1-9 FRI* over the TSS (C) and TTS (D) of genes upregulated in the *fld-4* compared to Col-0. (E) Quant-seq signal metaplot of Col*FRI* and *aprf1-9 FRI* over the TTS of genes commonly upregulated in both *aprf1-9 FRI* and *fld-4*. Venn diagrams were created with Venny (https://bioinfogp.cnb.csic.es/tools/venny/index.html). (F,G) Examples of non-coding transcripts upregulated in *aprf1-9 FRI* found by Quant-seq. (F) Quant-seq signal over the chromatin region of the protein-coding gene At2g06005 with indication in grey of the non-coding locus transcribed upstream in Col*FRI* (left) and *aprf1-9 FRI* (right). In the Col*FRI* sample we see the Quant-seq signal over the canonical polyadenylation sites of both the non-coding transcript and the protein-coding gene. In *aprf1-9 FRI* (right), we see 10-fold increase of the non-coding transcript signal and some non-canonical polyadenylation signals over the protein-coding gene, potentially coming from transcriptional interference and/or termination defects from transcribing the non-coding locus. (G) Similar plots showing the genomic environment of the At2g07040 protein coding gene. This locus has a convergently transcribed non-coding RNA. This non-RNA (At2g07042) is spliced and shows two main polyadenylation signals by Quant-seq on the wild-type strain (left). Similar to *COOLAIR* class III, in the mutant condition (right), there is a massive upregulation of intermediate isoforms of the transcript (purple arrow), potentially indicating a compromised proximal termination. Related to Figure 4.

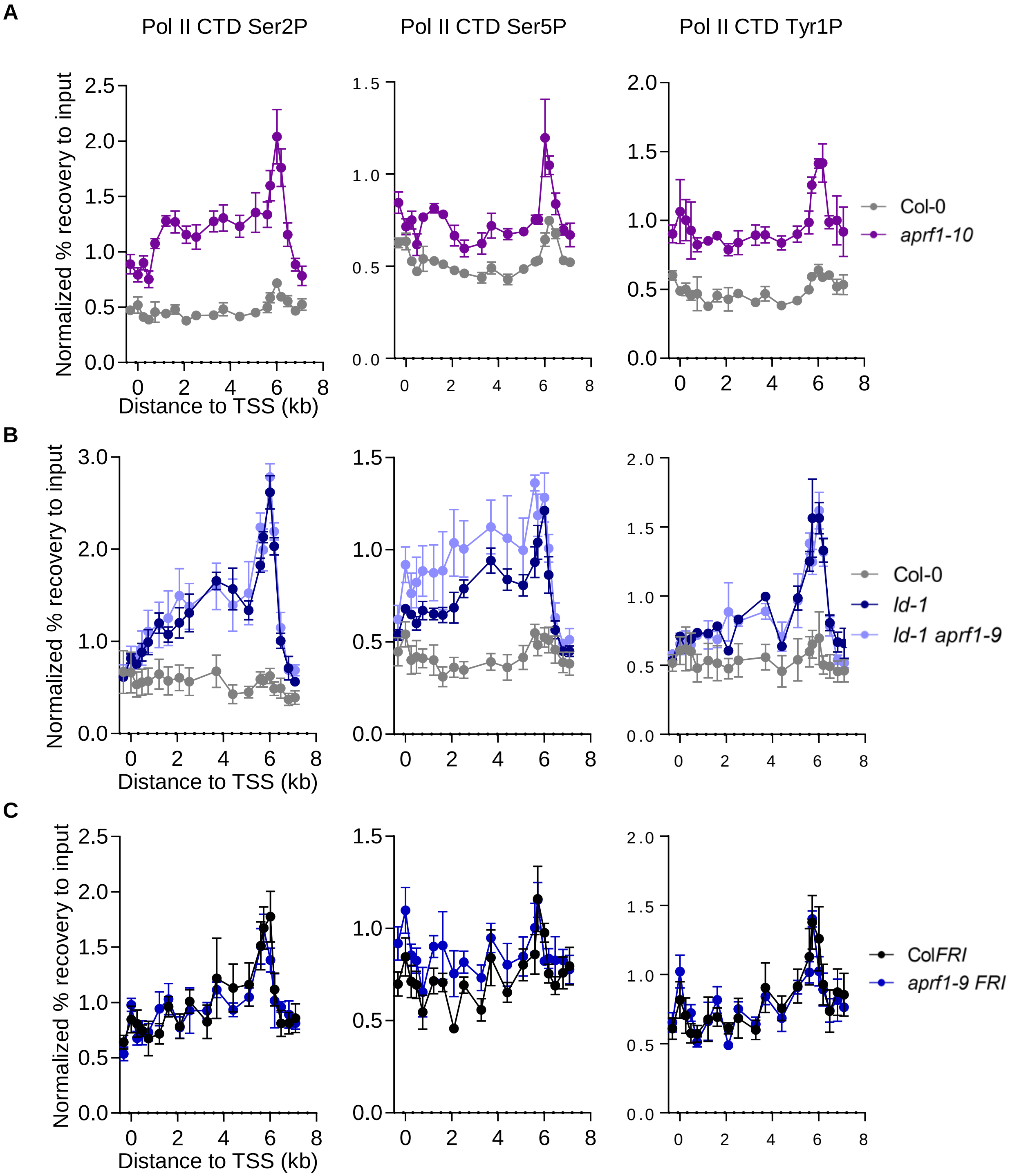

Figure S14. ChIP-qPCR levels of different phosphoisoforms of the RNA Pol II CTD. (A-C) Normalized percentage of Input recovery of the Ser2P (left), Ser5P (center), and Tyr1P (right) of Col-0 and *aprf1-10* (A), Col-0, *ld-1*, *aprf1-9 ld-1* (B), and Col*FRI* and *aprf1-9 FRI* (C). All the experiments were performed with 2-week-old seedlings. Dots and error bars represent average ± s.e.m. of three replicates. Each replicate consists in 2.5 gr of seedlings. Results were normalized as in 3A. X-axis represent the distance to TSS of *FLC* in kilobases (kb). Related to Figure 5.

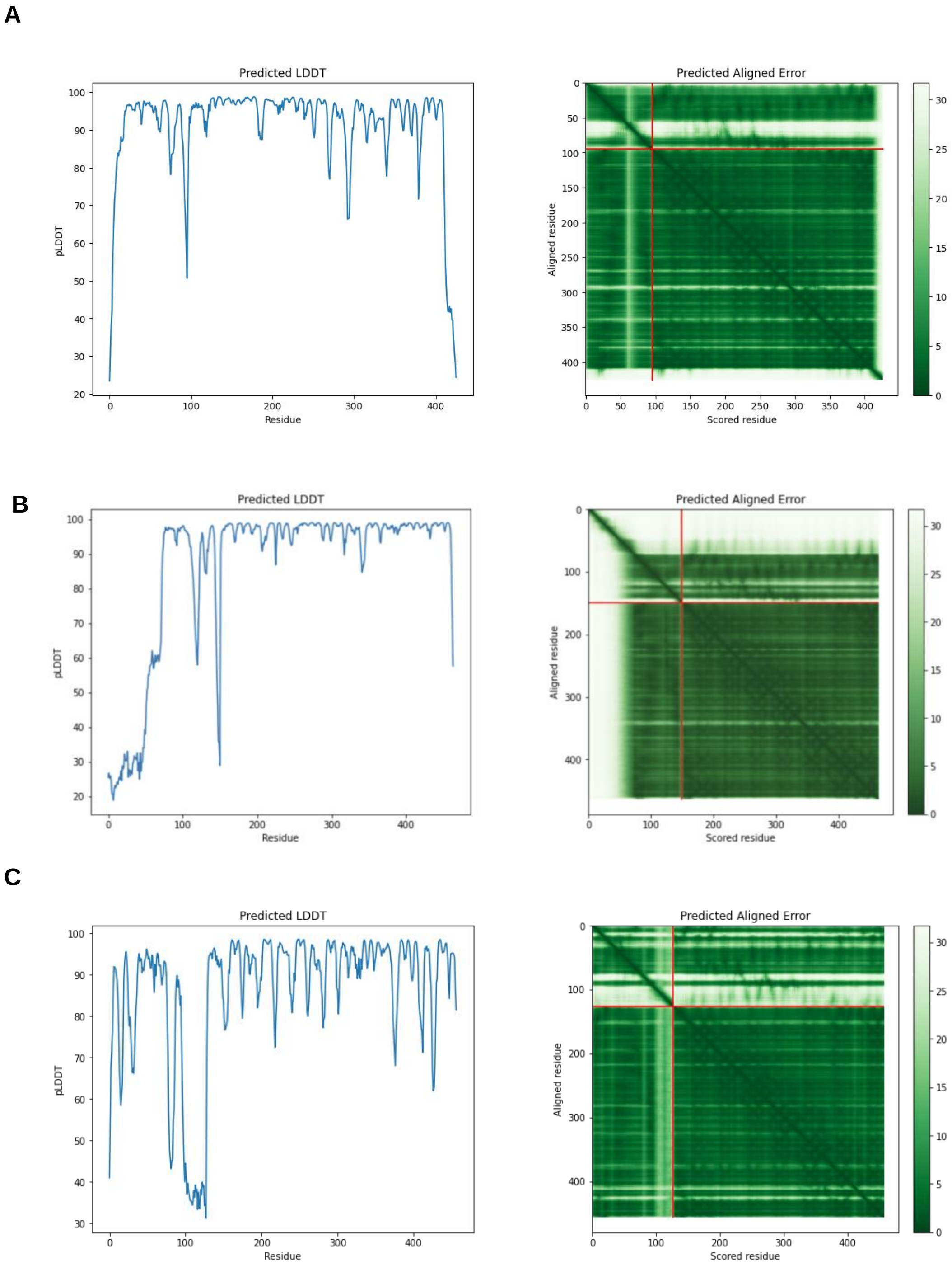

Figure S15. LDDT and PAE plots for AlphaFold2 predictions from Fig 2D. (A) Arabidopsis LD aa566-661 and APRF1 aa1-330, (B) human PNUTS aa380-530 and WDR82 aa1-313, (C) yeast Ref2 aa406-533 and Swd2 aa1-329 form well predicted complexes. Related to STAR Methods.

Table S4. Primer sets used in this work

Purpose	Oligonucleotide name	Oligonucleotide sequences (5' → 3')	
		Forward primer (F)	Reverse primer (R)
Genotyping	WiscDsLox489-492K11	GTTTTTCGAGCAAAGGGAAAG	ACAGGGTACACCCATAGAGGC
	LB_WiscDs		TCCTCGAGTTTCTCCATAATAATGT
	dCAPS_aprf1-10 (MaeII)		
	dCAPS_fca-9 (Sty1)	TGTTGAGATGGTGAAACTGTG	TCTTTGGCTCAGCAAACC
	fld-4	AGAAACCTGCCTGAATGTC	AGCTAGGCAACTGATGAG
	LBb1.3		ATTTTGCCGATTTCGGAAC
	ld-1	TGGTACAGGAGGATTGAATCG	CTCTGAACATCAGCCTCCTTG
	LB3		TAGCATCTGAATTTCATAACCAATCTC
	FRI	AGATTTGCTGGATTTGATAAGG	CTTGATGTTGGTCGATGATG
	35S:FCA	GCCACTTGCTCTCTCCTCAC	TTTCAATTCTCTCTGCCTCTCA
	s2lb	TGACAGCTAGCGACGATGAC	TGGTTGTAAGCAGCATGAGC
	LB (s2lb)		CCGGATCGTATCGGTTTTCG
Cloning	sgRNA_APRF1	AATGGGAGGGAAGATACATTCGGT	AAACACCGAATGTATCTTCCCTCC
	pKIR	AGAAGAGAAGCAGGCCCATT	AATGGATTGAGCCAAAGAGC
	APRF1-mVENUS	GGCCAGTGCCAAGCTAAGGATCGAAATTCCC<br>CCGAG	CGGCAGCAGAAAGCTTCTGTTGTTGATCGG<br>TAGGAGG
	TOPP4-3xFLAG	CCGGGTACCGAGCTCGAATTCCAAAAACGCA<br>ATTTAACATTTTTCCCATCA	CCTTATAGTCAATCTTTGTGGACATCATGA<br>ACTTGGT
RT	FLC spliced		TTTGTCCAGCAGGTGACATC
	FLC unspliced		CTTTGTAATCAAAGGTGGAGAGC
	UBC		TTGTGCCATTGAATTGAACCC
	PP2A		CCAAGCATGGCCGTATCATGT
	Total COOLAIR	TGCATCGAGATCTTGAGTGTATGT	
	Proximal COOLAIR	TGGTTGTTATTTGGTGGTGTG	

RT (continuation)	Distal COOLAIR	GCCCGACGAAGAAAAAGTAG	
	Class III COOLAIR	GAAACAATCTGGACAGTAGAGGC	
qPCR	FLC spliced	AGCCAAGAAGACCGAACTCA	TTTGTCCAGCAGGTGACATC
	FLC unspliced	CGCAATTTTCATAGCCCTTG	CTTTGTAATCAAAGGTGGAGAGC
	Proximal COOLAIR	CCTGCTGGACAAATCTCCGA	TCACACGAATAAGGTGGCTAATTAAG
	Distal COOLAIR	GTATCTCCGGCGACTTGAAC	GGATGCGTCACAGAGAACAG
	Total COOLAIR	TGCATCGAGATCTTGAGTGTATGT	ACGTCCCTGTTGCAAAATAAGC
	COOLAIR class III.1	AGTAGAGGCTTATGTTTAGGGTTCT	TCTCACACGAATAAGATTGAAAATGAC
	COOLAIR class III.2	AGGCTTATGTTTAGGGTTCTTATGTAC	TCCATCTGTACGATAATCATAGATTGA
	COOLAIR class III.3	GAAACAATCTGGACAGTAGAGGC	TGTCCAGCAGATTGAAAATGACA
	PP2A	ACTGCATCTAAAGACAGAGTTCC	CCAAGCATGGCCGTATCATGT
	UBC	CTGCGACTCAGGGAATCTTCTAA	TTGTGCCATTGAATTGAACCC
ChIP	FLC_-2285	ATCCAGAAAAGGGCAAGGAG	CGAATCGATTGGGTGAATG
	FLC_-1788	GGATTGATGTGGGGCACTAT	AGTCATGGGTAGGGCATGTG
	FLC_-1555	TGGAGGGAACAACCTAATGC	TCATTGGACCAAACCAAACC
	FLC_-321	ACTATGTAGGCACGACTTTGGTAAC	TGCAGAAAGAACCTCCACTCTAC
	FLC_5	GCCCGACGAAGAAAAAGTAG	TCCTCAGGTTTGGGTTCAAG
	FLC_246	CGACAAGTCACCTTCTCCAAA	AGGGGGAACAAATGAAAACC
	FLC_473	GGCGGATCTCTTGTTGTTTC	CTTCTTCACGACATTGTTCTTCC
	FLC_741	CGTGCTCGATGTTGTTGAGT	TCCCGTAAGTGCATTGCATA
	FLC_1212	CCTTTTGCTGTACATAAACTGGTC	CCAAACTTCTTGATCCTTTTTACC
	FLC_1613	TTGACAATCCACAACCTCAATC	TCAATTTCCTAGAGGCACCAA
	FLC_2094	AGCCTTTTAGAACGTGGAACC	TCTTCCATAGAAGGAAGCGACT
	FLC_2523	AGTTTGGCTTCCTCATACTTATGG	CAATGAACCTTGAGGACAAGG
	FLC_3276	GGGGCTGCGTTTACATTTTA	GTGATAGCGCTGGCTTTGAT
	FLC_3699	TGAAATGTTACGAATACTAGCGTGT	GGATCAAAACTACTAGCTAACCCTTG
	FLC_4406	AGAACAACCGTGCTGCTTTT	TGTGTGCAAGCTCGTTAAGC
	FLC_5090	CCGGTTGTTGGACATAACTAGG	CCAAACCCAGACTTAACCAGAC

	FLC_5599	TGGTTGTTATTTGGTGGTGTG	ATCTCCATCTCAGCTTCTGCTC
	FLC_5715	CCTGCTGGACAAATCTCCGA	GGATTTTGATTTCAACCGCCGA
ChIP	FLC_6013	CGTGTGAGAATTGCATCGAG	AAAAACGCGCAGAGAGAGAG
(continuation)	FLC_6189	TCCTAAACGCGTATGGTTGG	CCTTCATGGATGACGGAACT
	FLC_6480	TCCAGACGCCATTGTCATTA	AGAGTGCATTTTAACACTGACGA
	FLC_6810	TTGTAAAGTCCGATGGAGACG	ACTCGGCGAGAAAGTTTGTG
	FLC_7091	CATCGCTGTGTTATGCGTTT	GGCAAGTGTCGCAGTAAACA
	STM (for H3K27me3 normalization)	GCCCATCATGACATCACATC	GGGAACTACTTTGTTGGTGGTG
	ACT-914 (for RNA Pol II ChIP normalization)	TGGGTCTCATATAGAACACTCACAAAGGT	GACCAAAACCCGAATAGGAGCAAGA
	ACT_+122 (for H3K36me3 normalization)	CGTTTCGCTTTCCTTAGTGTTAGCT	AGCGAACGGATCTAGAGACTCACCTTG
	ACT+939 (for H3K4me1 normalization)	TGCCCCGAGAGCAGTGTTCC	TGGACTGAGCTTCATCACCAACG
